# Sepsis Prediction for the General Ward Setting

**DOI:** 10.3389/fdgth.2022.848599

**Published:** 2022-03-08

**Authors:** Sean C. Yu, Aditi Gupta, Kevin D. Betthauser, Patrick G. Lyons, Albert M. Lai, Marin H. Kollef, Philip R. O. Payne, Andrew P. Michelson

**Affiliations:** ^1^Institute for Informatics, Washington University School of Medicine in St. Louis, St. Louis, MO, United States; ^2^Department of Biomedical Engineering, Washington University in St. Louis, St. Louis, MO, United States; ^3^Department of Pharmacy, Barnes-Jewish Hospital, St. Louis, MO, United States; ^4^Division of Pulmonary and Critical Care, Washington University School of Medicine in St. Louis, St. Louis, MO, United States; ^5^Healthcare Innovation Lab, BJC HealthCare, and Washington University in St. Louis School of Medicine, St. Louis, MO, United States

**Keywords:** sepsis, electronic health records, machine learning, prediction, general ward

## Abstract

**Objective:**

To develop and evaluate a sepsis prediction model for the general ward setting and extend the evaluation through a novel pseudo-prospective trial design.

**Design:**

Retrospective analysis of data extracted from electronic health records (EHR).

**Setting:**

Single, tertiary-care academic medical center in St. Louis, MO, USA.

**Patients:**

Adult, non-surgical inpatients admitted between January 1, 2012 and June 1, 2019.

**Interventions:**

None.

**Measurements and Main Results:**

Of the 70,034 included patient encounters, 3.1% were septic based on the Sepsis-3 criteria. Features were generated from the EHR data and were used to develop a machine learning model to predict sepsis 6-h ahead of onset. The best performing model had an Area Under the Receiver Operating Characteristic curve (AUROC or c-statistic) of 0.862 ± 0.011 and Area Under the Precision-Recall Curve (AUPRC) of 0.294 ± 0.021 compared to that of Logistic Regression (0.857 ± 0.008 and 0.256 ± 0.024) and NEWS 2 (0.699 ± 0.012 and 0.092 ± 0.009). In the pseudo-prospective trial, 388 (69.7%) septic patients were alerted on with a specificity of 81.4%. Within 24 h of crossing the alert threshold, 20.9% had a sepsis-related event occur.

**Conclusions:**

A machine learning model capable of predicting sepsis in the general ward setting was developed using the EHR data. The pseudo-prospective trial provided a more realistic estimation of implemented performance and demonstrated a 29.1% Positive Predictive Value (PPV) for sepsis-related intervention or outcome within 48 h.

## Introduction

Sepsis is defined as a life-threatening organ dysfunction caused by a dysregulated host response to infection (1). In 2017, sepsis was responsible for 5.8% of all hospital stays and $38.2 billion in hospital costs (2). Moreover, sepsis has a high mortality rate and was found to be implicated in about one in every three inpatient deaths (3).

Early and effective therapy is critical in the management of patients with sepsis, as prolonged recognition and delayed treatment increase mortality (4, 5). As a result, there is an abundance of literature focusing on the early detection and prediction of sepsis through traditional or newly developed scoring systems such as the Systemic Inflammatory Response Syndrome (SIRS) score, National Early Warning Score (NEWS), or quick Sequential Organ Failure Assessment (qSOFA) score; or more recently through the use of machine learning models (6–8). Most of these efforts focus on the Emergency Department (ED) or Intensive Care Unit (ICU) settings which are data-rich and have a higher prevalence of sepsis compared to the general ward setting (9–11). However, patients who develop sepsis in the general ward setting have worse outcomes compared to those who develop sepsis in the ED or ICU (12). Because general ward patients are observed less closely than in the ED or ICU setting with fewer vital signs documented and laboratory tests performed, they represent a proportionally more vulnerable population that could benefit more from an augmented sepsis early warning system. Therefore, the objective of this study was to develop a machine learning model for predicting sepsis in the general ward setting, compare its performance to commonly used instruments for sepsis surveillance such as SIRS and NEWS, and extend the model evaluation using a novel simulated pseudo-prospective trial (13, 14).

## Materials and Methods

### Study Design, Data Sources, and Population

The model was developed and validated using the Electronic Health Record (EHR) data from Barnes-Jewish Hospital / Washington University School of Medicine in St. Louis, a large, academic, tertiary-care academic medical center. All patients ≥18 years of age that were admitted to the hospital between January 1, 2012 and June 1, 2019 were eligible for inclusion. Patients were excluded if they were admitted to the Psychiatry or Obstetrics services, due to highly variable rates of physiologic data collection. Encounters were excluded if there were no billing code, vital signs, laboratory, service, room, or medication data to indicate a complete patient stay. Encounters were also excluded if the total length of stay was below 12 h or exceeded 30 days. After the assignment of index time and prediction time, further exclusion criteria were applied based on that index time (Supplementary Methods 3). To focus on patients most likely to benefit from a risk prediction model, the following populations were excluded: patients who had cultures procured or received antibiotics within 48 h prior to prediction time; patients who had sepsis present on admission (by admission International Classification of Diseases (ICD) code); patients who were in the ICU within 24 h prior to the prediction time. To avoid the conflation of post-surgical care with sepsis care, patients were ineligible if they had surgery within 72 h prior to the prediction time. To avoid predicting on patients with excessive missingness, encounters were also required to have at least 3 of each vital sign and at least one complete blood count, and one basic or comprehensive metabolic panel test within 24 h prior to the prediction time (Supplementary Figure 1).

This project was approved with a waiver of informed consent by the Washington University in St. Louis Institutional Review Board (IRB #201804121).

### Sepsis Definition

Sepsis was defined using the Sepsis-3 implementation based on Suspicion of Infection (SOI) determined by concomitant antibiotics and cultures, Sequential Organ Failure Assessment (SOFA) score in the ICU setting, and qSOFA elsewhere (Supplementary Methods 3) (8, 15). The anti-infectives for SOI was limited to intravenous anti-infectives except oral vancomycin and metronidazole. In accordance with the Sepsis-3 criteria, SOI required either having antibiotics within 72 h of culture collection or culture collection within 24 h of having antibiotics (8). The time of Suspicion of Infection (T_SOI_) was the time either before antibiotic order start time or culture collection time (15). To meet sepsis criteria, the patient must have had a SOFA or qSOFA score ≥ 2, depending on the location, between 48 h prior to and 24 h after T_SOI_. For sepsis cases, the time of sepsis onset (T_Sepsis_) was the same as T_SOI_.

To facilitate the model development, each encounter was assigned an index time (T_Index_), which for sepsis encounters was T_Sepsis_, and for non-sepsis encounters was 6-h prior to the maximum of either (1) the midpoint between admission and discharge, or (2) 12 h into admission. The time of prediction (T_Prediction_) was 6-h prior to the index time (**eMethods 3**).

### Feature Generation and Engineering

Features were generated from the demographics, locations, medications, vital signs, and laboratory data available until the time of prediction (Supplementary Methods 2). Medications were mapped to classes and subcategories of the Multum MediSource Lexicon by Cerner (Denver, CO). Comorbidities were determined using ICD codes only from prior admissions and were mapped using the Elixhauser comorbidity system (16). The time series data were summarized as various univariate statistics (max, mean, etc.) over multiple time horizons (3 h, 6 h, etc.). Measures of variance such as SD were only computed if there were at least 4 measurements within the time horizon. Missing values, especially results of non-routine lab tests, were likely not missing at random but as a result of clinical judgment, thus, were kept as is (Supplementary Table 2). Features with >75% missingness, however, were excluded as they are unlikely to improve performance. For models that required fully non-null input, mean-imputation was used.

### Model Development

Patient encounters were split at the patient level to avoid “identity confounding” into the train (75%) and test sets (25%) (17). Data transformation parameters were generated based on the training set, then applied to both sets. Random search with repeated cross-validation on the training set was used to tune the hyperparameters of an eXtreme Gradient Boosting (XGBoost) model, and the optimal combination was used for training on the full training set (Supplementary Methods 4) (18). The feature importance for the optimized XGBoost model (**XGB opt**) was estimated using the well-validated SHAP approach, a method of credit attribution based on coalitional game theory with useful properties such as additivity and the ability to provide explanations for individual predictions (19). To condense the model into one that is easier to transport and implement, a “lite” version of the XGBoost model (**XGB lite**) was created using a small subset of features based on the sum of the absolute SHapley Additive exPlanations (SHAP) values across the training set (Supplementary Figure 2). For comparison, an XGBoost model with default parameters (**XGB unopt**) was trained, as was a logistic regression model with l2 regularization (**LogReg**; Supplementary Figure 3).

### Model Performance

The trained models were compared against the **SIRS** score, National Early Warning Score 2 (**NEWS2**), and **qSOFA** score (6–8, 20). Using the data from within the 24-h time window preceding prediction time, SIRS was calculated as the highest score occurring within a 1-h sliding window; NEWS2 was calculated using the last available measurements; qSOFA was calculated using the most abnormal measurements. For SIRS, NEWS2, and qSOFA, the lack of measurements was interpreted as normal.

The performance of the model was evaluated on bootstrap samples of the test set. Evaluated metrics include the Area Under the Receiver Operating Characteristic curve (**AUROC**) and the Area Under the Precision-Recall Curve (**AUPRC**). Model calibration was assessed by binning the test set into deciles of predicted risk and comparing their predicted probability of sepsis with the actual proportion of sepsis cases. The impact of threshold selection was visualized by plotting performance metrics (specificity, sensitivity, etc.) against the probability threshold.

### Pseudo-Prospective Trial

While the model was trained and evaluated on a single time point per encounter, real-world implementation would likely involve continuous risk prediction throughout patient encounters. To better understand the implemented performance of the best performing sepsis prediction algorithm, the model was applied hourly to patient encounters in the test set spanning full admission duration. Patients whose model prediction crossed the threshold maximizing F1 score (harmonic mean of precision and recall) will hereby be referred to as having been “alerted on,” and for those, “alert time” was defined as the first alert instance for the encounter. For each patient hour, time-sensitive exclusion criteria (e.g., not in the general ward or already on anti-infectives) were applied again to remove inappropriate alerts. First, the cross-tabulation of sepsis status and alert status was generated. Then, among those who were alerted, we assessed the proportion of encounters with the following sepsis-related interventions and outcomes: sepsis-relevant culture collection, sepsis-relevant anti-infective administration, ventilator initiation, ICU transfer, sepsis onset, or death.

### Statistical Analysis

Variables were summarized using frequencies and proportions for categorical data or medians and interquartile ranges (IQR) for continuous data. Statistical comparisons were performed using the Chi-square and Mann–Whitney *U* tests where appropriate. A *p*-value < 0.01 was considered statistically significant. Analysis and figure generation were performed with Python version 3.7.1 (Python Software Foundation, Beaverton, OR) using the following packages: scipy, numpy, pandas, matplotlib, sklearn, xgboost, and shap (18, 21–26).

## Results

### Patient Population

From the initial inpatient population of 401,235 encounters, 331,201 met exclusion criteria, leaving 70,034 encounters in the final cohort (Supplementary Figure 1). Application of the Sepsis-3 criteria identified 2,206 (3.1%) patient with sepsis encounters. Patients with sepsis were slightly older [65.6 (56.3–74.3) vs. 60.8 (49.4–71.2), *p* < 0.01], more likely to be white (71.3 vs. 61.8%, *p* < 0.01), had a higher Elixhauser comorbidity score [19 (10–29) vs. 9 (1–17), *p* < 0.01], a longer length of stay [12.9 (8.0–19.3) vs. 3.9 (2.3–6.7), *p* < 0.01], and higher inpatient mortality (16.6% vs. 0.8%, *p* < 0.01) (Table 1, Supplementary Table 3).

**Table 1:** Cohort characteristics.

**Variable**	**Total** **[*n* = 70,034** **(100.0%)]**	**Sepsis** **(*n* = 2,206 [3.1%])**	**Non-sepsis** **(*n* = 67,828 [96.9%])**	**p^a^**
				<0.01*
Age (years), median (IQR)	61.0 (49.6–71.3)	65.5 (56.3–74.3)	60.8 (49.4–71.2)	<0.01*
Sex (female), *n* (%)	32,751 (46.8%)	992 (45.0%)	31,759 (46.8%)	0.090
Race, *n* (%)				<0.01*
White, *n* (%)	43,516 (62.1%)	1,573 (71.3%)	41,943 (61.8%)	<0.01*
Other/unknown, *n* (%)	3,787 (5.4%)	129 (5.8%)	3,658 (5.4%)	0.378
Black, *n* (%)	22,285 (31.8%)	487 (22.1%)	21,798 (32.1%)	<0.01*
Asian, *n* (%)	446 (0.6%)	17 (0.8%)	429 (0.6%)	0.505
BMI, median (IQR)	27.6 (23.5–33.0)	27.2 (23.1–33.4)	27.6 (23.5–33.0)	0.252
Admitted through ED, *n* (%)	33,364 (47.6%)	747 (33.9%)	32,617 (48.1%)	<0.01*
LOS (days), median (IQR)	3.9 (2.4–7.0)	12.9 (8.0–19.3)	3.9 (2.3–6.7)	<0.01*
Discharge disposition				<0.01*
Home, *n* (%)	59,367 (84.8%)	1,185 (53.7%)	58,182 (85.8%)	<0.01*
Hospice, *n* (%)	854 (1.2%)	88 (4.0%)	766 (1.1%)	<0.01*
Acute care facility, *n* (%)	436 (0.6%)	17 (0.8%)	419 (0.6%)	0.447
Nonacute care facility, *n* (%)	8,234 (11.8%)	539 (24.4%)	7,695 (11.3%)	<0.01*
In-hospital death, *n* (%)	889 (1.3%)	367 (16.6%)	522 (0.8%)	<0.01*
Other, *n* (%)	254 (0.4%)	10 (0.5%)	244 (0.4%)	0.589
Sepsis discharge ICD code^b^				<0.01*
Sepsis, *n* (%)	1,049 (1.5%)	543 (24.6%)	506 (0.7%)	<0.01*
Severe sepsis, *n* (%)	510 (0.7%)	358 (16.2%)	152 (0.2%)	<0.01*
Septic shock, *n* (%)	378 (0.5%)	293 (13.3%)	85 (0.1%)	<0.01*
30-day readmission, *n* (%)	14,817 (21.2%)	440 (19.9%)	14,377 (21.2%)	0.165
Elixhauser comorbidity score, median (IQR)^c^	9 (1–18)	19 (10–29)	9 (1–17)	<0.01*

*BMI, body mass index; ED, emergency department; LOS, length of stay; ICD, International Classification of Diseases*.

a Comparison of variables between sepsis and non-sepsis cohort was performed using Mann–Whitney U test for continuous variables, and χ^2^ for categorical variables. Statistical significance (p < 0.01) is denoted by *.

b *Based on sepsis discharge ICD code list from (27)*.

c *Based on Elixhauser comorbidity weights from (28)*.

### Model Performance

**Figure 1:**
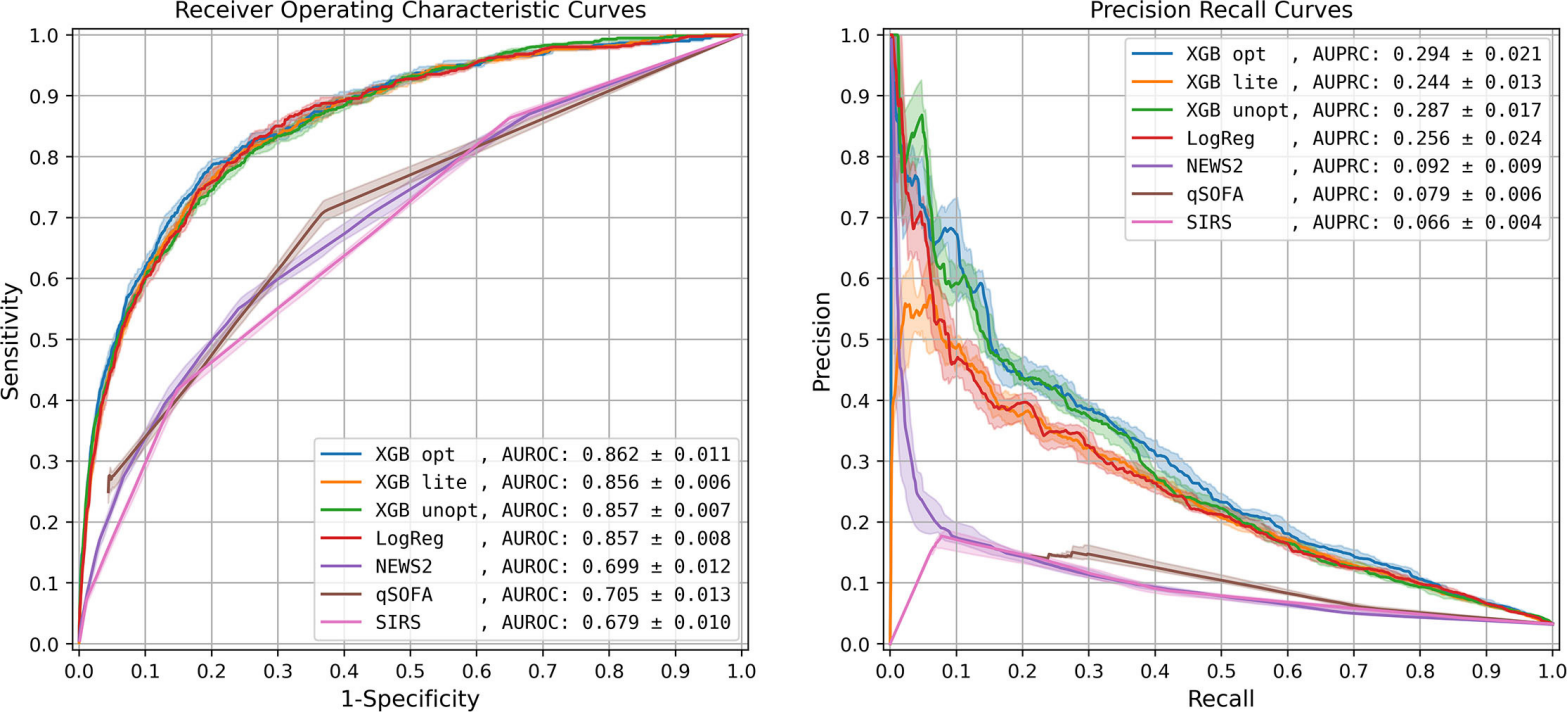
Model performance: Receiver Operating Characteristic curve and Precision-Recall curve. The solid lines represent the 50th percentile curves based on 20 bootstraps (full resampling with replacement) iterations of the test dataset, and the shaded regions represent the area between the 25th and 75th percentiles. AUROC, area under receiver operating characteristic curve; AUPRC, area under precision recall curve; XGB opt, optimized XGBoost model; XGB lite, simple XGBoost model; XGB unopt, unoptimized, out-of-the-box XGBoost model; LogReg, logistic regression; NEWS2, National Early Warning Score 2; qSOFA, quick Sequential Organ Failure Assessment; SIRS, Systemic Inflammatory Response Syndrome.

The optimized XGBoost model (**XGB opt**) using all 1,071 features had the highest AUROC (0.862 ± 0.011) and AUPRC (0.294 ± 0.021), compared to the unoptimized XGBoost model (**XGB unopt**), logistic regression (**LogReg**), and the lite XGBoost model (**XGB lite**), all of which had similar performances only slightly worse than XGB opt (Figure 1, Supplementary Table 4). The scoring systems, however, had a significantly lower performance with a loss in AUROC over 0.150.

**Figure 2:**
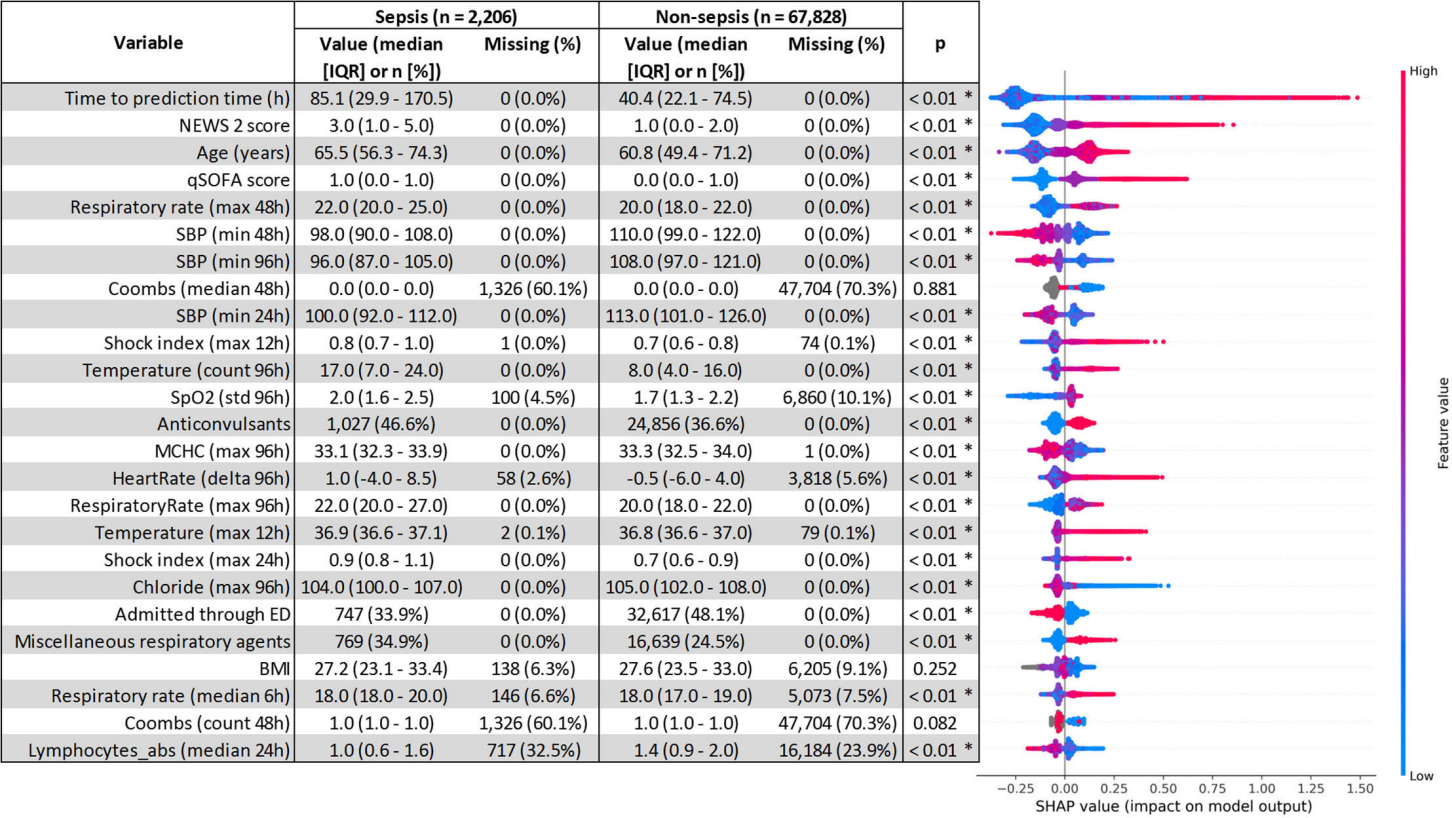
SHapley Additive exPlanations (SHAP) feature importance. Comparison of variables between the sepsis and non-sepsis cohort was performed using the Mann–Whitney *U* test for continuous variables, and χ^2^ for categorical variables. Statistical significance (*p* < 0.01) is denoted by *. qSOFA, quick sequential organ failure assessment; NEWS2, national early warning system 2; SBP, systolic blood pressure; WBC, white blood cell count; MAP, mean arterial pressure.

**Figure 3:**
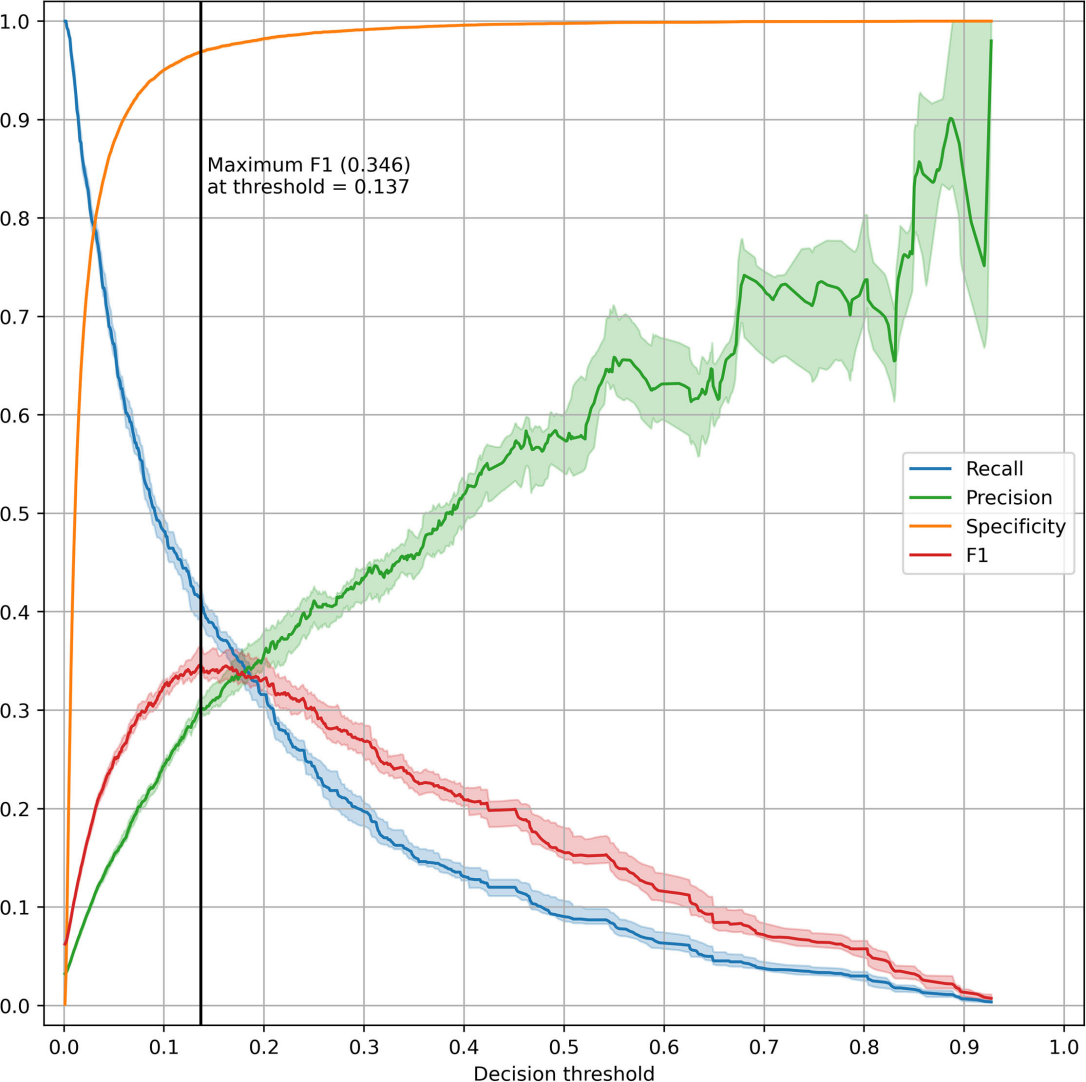
Threshold plot for the optimized XGBoost model. The test set was bootstrapped (full resampling with replacement) 20 times and various performance metrics (recall, precision, specificity, and F1) were plotted against the threshold value. For each metric, the line and shaded area represent the median and IQR. A vertical black line was drawn at the threshold maximizing the F1 score.

The top five most impactful features for the optimized XGBoost model were found to be: time from admission to prediction time, NEWS2 score, age, qSOFA score, and maximum respiratory rate within 48 h prior to prediction time (Figure 2). The calibration curve yielded an *r*^2^ value of 0.837 (Supplementary Figure 4). The threshold plot demonstrates the tradeoff between precision and recall and revealed the highest F1 score (0.346) to be at a threshold of around 0.137 (Figure 3).

### Pseudo-Prospective Trial

The EHR data of the 17,441 encounters in the test set (557 sepsis encounters and 16,884 non-sepsis encounters) were binned hourly into 2,387,482 patient hours. After exclusions, 3,532 encounters were alerted upon, of which 388 met the sepsis criteria (11.0% PPV) (Supplementary Table 4). Of the 557 sepsis encounters, 388 were alerted upon (69.7% sensitivity). Of the 13,740 non-sepsis encounters, 3,144 were alerted upon (81.4% specificity).

Of the 3,532 alerted encounters, from the time of the first alert, within 48 h, 23.9% had sepsis-relevant cultures drawn, 13.2% received sepsis-relevant anti-infectives, 2.5% had ventilator initiated, 6.9% experienced sepsis onset, 4.7% were transferred to ICU, and 0.6% died (Table 2, Supplementary Table 5). Altogether, 29.1% experienced a sepsis-related intervention or outcome within 48 h of the first alert.

**Table 2:** Pseudoprospective trial, outcomes for alerted subjects.

**Intervention or outcome**	**Within 24 h**	**Within 48 h**	**Within 72 h**
Sepsis-relevant cultures	600 (17.0%)	843 (23.9%)	1,018 (28.8%)
Sepsis-relevant anti-infectives	286 (8.1%)	466 (13.2%)	591 (16.7%)
Ventilator initiation	51 (1.4%)	87 (2.5%)	119 (3.4%)
Sepsis onset	182 (5.2%)	245 (6.9%)	291 (8.2%)
ICU transfer	112 (3.2%)	167 (4.7%)	209 (5.9%)
Death	8 (0.2%)	21 (0.6%)	36 (1.0%)
Total	739 (20.9%)	1,028 (29.1%)	1,237 (35.0%)

*Of the patients who crossed the set threshold in the pseudoprospective trial, and of those who were not already suspected of or being treated for sepsis, sepsis-related interventions and outcomes within various time horizons were identified*.

Visualizations of the sample patient trajectories alongside hourly predicted sepsis risk scores facilitated inspections of model successes and failures (Supplementary Figure 5).

## Discussion

The objective of this study was to develop a machine learning model capable of predicting sepsis 6-h ahead of clinical onset using one of the largest inpatient EHR datasets. Unlike most sepsis prediction studies which focus on the data-rich ICU or ED setting, this study focused on the general ward setting where the prediction task is made especially challenging due to the sparsity of data and low prevalence (29). Moreover, the cohort criteria excluded patients who were already suspected of, or were being treated for sepsis, as a clinical prediction model is unlikely to benefit these patients. The resultant cohort represents patients who were not captured by clinical judgment and thus could benefit from clinical decision support. Further, this study provides a novel way of better estimating real-world performance through the assessment of a pseudo-prospective trial.

Excluding patients who were suspected of or were already being treated for sepsis, alongside several other exclusion criteria, resulted in the elimination of the majority of inpatients from the initial population (Supplementary Figure 1). As a result, the retained sepsis cohort are likely cases of hospital-acquired sepsis or community-acquired sepsis with delayed recognition. Though the restrictive exclusion criteria may limit generalizability, the resultant cohort is more likely to benefit from an automated warning system.

We compared the performance of several machine learning models as well as traditional scoring systems and found the optimized XGBoost to have the best AUROC and AUPRC for detecting sepsis ahead of meeting traditional diagnostic criteria. The “lite” model was used with 25 features and had a similar diagnostic performance.

Of the important features, as determined by SHAP, time from admission to prediction time was the most important, indicating that prolonged length of stay is both a risk factor and outcome for sepsis. The qSOFA and NEWS2 scores were also important predictors, demonstrating the utility of these scores as features though deficient on their own. Admission through the ED was associated with a lower probability of sepsis, likely due to the emphasis on sepsis screening in the ED setting. Interestingly, while most medication information was not important for the model, anticonvulsants had a surprisingly high SHAP value with sepsis patients receiving “anticonvulsants” about 10% more frequently than non-sepsis patients (46.6 vs. 36.6%, Figure 2). However, the Multum classification for anticonvulsants included medications such as magnesium sulfate and lorazepam which are not always used as anticonvulsants, thus, more work is needed on automated feature generation from medication data. Another unexpectedly important feature was the Coombs test, which is unlikely to be related to sepsis, but had noticeably different rates of missingness between sepsis and non-sepsis patients (60.1% for sepsis vs. 70.3% for non-sepsis, Figure 2). Comorbidities from prior admissions were noticeably absent from the list of important features, likely because 46.3% of all encounters were first encounters and did not have any prior admissions. It's possible that the importance of comorbidities as features may rise with time, with larger populations with longer histories being collected in the electronic health record and the ability to retrieve information cross-sites.

The pseudo-prospective trial demonstrated a novel approach to better estimating real-world model performance and showed that 29.1% of the alerted patients required sepsis-related intervention or had a sepsis-related outcome within 48 h (Table 2). While the algorithm was capable of identifying patients who ultimately required cultures (39.0%) and anti-infectives (28.1%), the actual incidence of Sepsis-3 onset after the patients were alerted on was relatively low (11.0% at any point after and 6.9% within 48 h). This may be due to problems in labeling—despite our attempt to exclude surgical patients from the cohort, they are not capable of being excluded on a prospective basis and frequently meet the sepsis criteria. Moreover, alerted patients may be critically ill and treated for sepsis but not meet the Sepsis-3 criteria. Also, many patients who are in the ED have higher scores which improve through interventions, but then have scores that rise again later during their stay at the hospital. Since this only evaluated the first time a patient crossed the sepsis threshold, the subsequent and potentially more important clinical changes would be missed. The pseudo-prospective trial highlights some of the anticipated challenges of translating a diagnostic scoring method from a retrospective data set to a prospective population, which necessitates further investigation.

Impressively, the unoptimized XGBoost solution had a median AUROC just 5% lower than the optimized version, and similar performance to the optimized logistic regression model and the lite XGBoost model. The relatively small benefit conferred by the more complex model compared to logistic regression is consistent with prior literature (30). If the added complexity is problematic—for interpretability, debugging, or implementation—then it could be argued that the simpler logistic regression model is preferred despite the performance loss. Though NEWS2 and qSOFA were very important features in XGBoost, the gap between traditional scoring systems and machine learning models was noticeable with the worst ML model conferring a 15.1% AUROC improvement over the best traditional scoring system.

This study has limited generalizability as a single institution study. The study used an interpretation of the Sepsis-3 definition and is likely to generalize poorly to sites using alternate definitions (1, 8, 15). By design, the study was focused on the general ward setting, and the results are not applicable to other settings. Many of the excluded subpopulations (children, surgical, etc.) warrant further investigation. While a pseudo-prospective trial was performed, a true prospective study is needed to gauge real-world performance. The pseudo-prospective trial could be further improved by investigating repeated alerts, incorporating alert lock-out periods, accounting for measurement-to-documentation time gap, etc. For the pseudo-prospective trial, a threshold was assigned to maximize the F1 score. However, further work is necessary to define an operationally meaningful threshold. For the calculation of the qSOFA score, GCS was missing in our dataset and assumed normal, which may negatively impact the sepsis label assignment process. However, Seymour et al. found that the lack of GCS in the VA dataset did not significantly reduce the predictive validity of qSOFA (8). As is typical of studies using electronic health records data, there were and likely remain problems concerning missingness and accuracy of clinical data.

## Conclusion

A machine learning model designed to predict sepsis 6-h ahead of meeting diagnostic criteria yielded an AUROC of 0.862 ± 0.011 and an AUPRC of 0.294 ± 0.021. Pseudo-prospective evaluation of the model revealed relatively good clinical performance, despite a large class imbalance.

## Data Availability Statement

The data analyzed in this study is subject to the following licenses/restrictions: The dataset includes protected health information, specifically electronic health records with patient identifiers, thus cannot be shared. Requests to access these datasets should be directed to i2help@wustl.edu.

## Ethics Statement

The studies involving human participants were reviewed and approved by Washington University Institutional Review Board #201804121. The Ethics Committee waived the requirement of written informed consent for participation.

## Author Contributions

AM, AG, and SY contributed to study conception and design as well as data collection. Primary analysis of data was performed by SY, the results of which were discussed with AM, KB, and AG. The manuscript was drafted by SY and AM. The manuscript was reviewed and approved by all authors.

## Funding

This work was supported by the Big Ideas Grant **(**Washington University School of Medicine Institute for Informatics and Healthcare Innovation Lab).

## Conflict of Interest

The authors declare that the research was conducted in the absence of any commercial or financial relationships that could be construed as a potential conflict of interest.

## Publisher's Note

All claims expressed in this article are solely those of the authors and do not necessarily represent those of their affiliated organizations, or those of the publisher, the editors and the reviewers. Any product that may be evaluated in this article, or claim that may be made by its manufacturer, is not guaranteed or endorsed by the publisher.
