# Supplementary material for: Sepsis Prediction for the General Ward Setting
Source: Front Digit Health. 2022 Mar 8;4:848599. doi: 10.3389/fdgth.2022.848599 (PMC8957791; doi:10.3389/fdgth.2022.848599)
Supplement: Supplementary file 1 [file Data_Sheet_1.docx]

Digital Supplemental Content

eMethods, eTables, and eFigures

[eTable 1. TRIPOD checklist 2](#_Toc78449531)

[eMethods 1. Data source 4](#_Toc78449532)

[eMethods 2. Data preprocessing and mapping 5](#_Toc78449533)

[eTable 2. Data availability 6](#_Toc78449534)

[eMethods 3. Index time identification for sepsis and non-sepsis cohorts 9](#_Toc78449535)

[eFigure 1. Cohort selection PRISMA-style diagram 10](#_Toc78449536)

[eTable 3. Cohort characteristics: comorbidities 11](#_Toc78449537)

[eMethods 4. XGBoost hyperparameter optimization 12](#_Toc78449538)

[eFigure 2. Feature selection for lite model 17](#_Toc78449539)

[eFigure 3. Logistic regression hyperparameter optimization 18](#_Toc78449540)

[eTable 4. Model performance comparison 19](#_Toc78449541)

[eFigure 4. Calibration plot for optimized XGBoost model 20](#_Toc78449542)

[eTable 4. Pseudo-prospective trial, alert confusion matrix 21](#_Toc78449543)

[eTable 5. Pseudo-prospective trial, time to intervention or outcome for alerted subjects 22](#_Toc78449544)

[eFigure 5. Pseudoprospective trial, patient trajectory visualizations 23](#_Toc78449545)

# eTable 1. TRIPOD checklist

| **Section/Topic** | **Item** | **Checklist Item** | **Page** |
| --- | --- | --- | --- |
| **Title and abstract** | | | |
| Title | 1 | Identify the study as developing and/or validating a multivariable prediction model, the target population, and the outcome to be predicted. | Title |
| Abstract | 2 | Provide a summary of objectives, study design, setting, participants, sample size, predictors, outcome, statistical analysis, results, and conclusions. | Abstract |
| **Introduction** | | | |
| Background and objectives | 3a | Explain the medical context (including whether diagnostic or prognostic) and rationale for developing or validating the multivariable prediction model, including references to existing models. | Introduction, paragraph 1 & 2 |
|  | 3b | Specify the objectives, including whether the study describes the development or validation of the model or both. | Introduction, paragraph 2 |
| **Methods** | | | |
| Source of data | 4a | Describe the study design or source of data (e.g., randomized trial, cohort, or registry data), separately for the development and validation data sets, if applicable. | Methods, “Study Design, Data Sources, and Population” section, and eMethods 1 |
|  | 4b | Specify the key study dates, including start of accrual; end of accrual; and, if applicable, end of follow-up. | Methods, “Study Design, Data Sources, and Population” section |
| Participants | 5a | Specify key elements of the study setting (e.g., primary care, secondary care, general population) including number and location of centres. | Methods, “Study Design, Data Sources, and Population” section |
|  | 5b | Describe eligibility criteria for participants. | Methods, “Study Design, Data Sources, and Population” section |
|  | 5c | Give details of treatments received, if relevant. | N/A |
| Outcome | 6a | Clearly define the outcome that is predicted by the prediction model, including how and when assessed. | Methods, “Sepsis Definition” section and eMethods 3 |
|  | 6b | Report any actions to blind assessment of the outcome to be predicted. | The outcome was determined in an automated fashion using consensus criteria definition, methods, “Sepsis Definition” section and eMethods 3 |
| Predictors | 7a | Clearly define all predictors used in developing or validating the multivariable prediction model, including how and when they were measured. | Methods, “Feature Generation and Engineering” section |
|  | 7b | Report any actions to blind assessment of predictors for the outcome and other predictors. | The predictors were extracted in an automated fashion, methods, “Feature Generation and Engineering” section |
| Sample size | 8 | Explain how the study size was arrived at. | Methods, “Study Design, Data Sources, and Population” section and results, “Patient Population” section |
| Missing data | 9 | Describe how missing data were handled (e.g., complete-case analysis, single imputation, multiple imputation) with details of any imputation method. | Methods, “Feature Generation and Engineering” section |
| Statistical analysis methods | 10a | Describe how predictors were handled in the analyses. | Methods, “Feature Generation and Engineering” section |
|  | 10b | Specify type of model, all model-building procedures (including any predictor selection), and method for internal validation. | Methods, “Feature Generation and Engineering”, “Model Development”, and “Model Performance” sections |
|  | 10d | Specify all measures used to assess model performance and, if relevant, to compare multiple models. | Methods, “Model Performance” and “Pseudo-Prospective Trial” sections |
| Risk groups | 11 | Provide details on how risk groups were created, if done. | N/A |
| **Results** | | | |
| Participants | 13a | Describe the flow of participants through the study, including the number of participants with and without the outcome and, if applicable, a summary of the follow-up time. A diagram may be helpful. | Methods, “Study Design, Data Sources, and Population” section; Results, “Patient Population” section, and eFigure 1 |
|  | 13b | Describe the characteristics of the participants (basic demographics, clinical features, available predictors), including the number of participants with missing data for predictors and outcome. | Results, “Patient Population” section, Table 1, eTable 2 |
| Model development | 14a | Specify the number of participants and outcome events in each analysis. | Results, “Patient Population” section, Table 1, eTable 2 |
|  | 14b | If done, report the unadjusted association between each candidate predictor and outcome. | Elided because too many. Subset shown in eFigure 2. |
| Model specification | 15a | Present the full prediction model to allow predictions for individuals (i.e., all regression coefficients, and model intercept or baseline survival at a given time point). | Elided because too many |
|  | 15b | Explain how to the use the prediction model. | Discussion |
| Model performance | 16 | Report performance measures (with CIs) for the prediction model. | Bootstrapped mean and standard deviation reported in Figure 1 and eTable 4 |
| **Discussion** | | | |
| Limitations | 18 | Discuss any limitations of the study (such as nonrepresentative sample, few events per predictor, missing data). | Discussion, last paragraph |
| Interpretation | 19b | Give an overall interpretation of the results, considering objectives, limitations, and results from similar studies, and other relevant evidence. | Results and Discussion |
| Implications | 20 | Discuss the potential clinical use of the model and implications for future research. | Discussion |
| **Other information** | | | |
| Supplementary information | 21 | Provide information about the availability of supplementary resources, such as study protocol, Web calculator, and data sets. | Supplementary digital content referred to and linked throughout manuscript |
| Funding | 22 | Give the source of funding and the role of the funders for the present study. | Title page, “Financial Support” section |

# eMethods 1. Data source

The data for analysis was sourced from Barnes-Jewish Hospital (BJH), one of the fifteen hospitals owned by BJC Healthcare, a non-profit health care organization based in St. Louis, MO and affiliated with Washington University in St. Louis, St. Louis, MO. During the time period from which the data was extracted, BJH primarily used the COMPASS EHR (Allscripts Sunrise, Chicago, IL). Clinical data was first loaded into to a hospital-managed data warehouse called Health Data Core (HDC), which is primarily used for quality improvement, then was loaded into to a university-managed research data warehouse called Research Data Core (RDC). All relevant data for inpatients between 1/12012 and 6/1/2019 was extracted from RDC.

# eMethods 2. Data preprocessing and mapping

Raw clinical data were mapped to cogent clinical concepts through a combination of informatics approaches and subject matter expert manual review.

Certain data elements were not present or partially present, but were able to be derived from related data elements:

- BMI = weight (kg) / (height (m))^2^. BMI was explicitly present for 35.3% of the study population, was able to be calculated for 91.8%, and was ultimately available for 92.0%.
- FiO2 was available explicitly, but was also calculated whenever there was oxygen flow documentation according to the following formula: oxygen flow x 3.5 + 21.
- PaO2 - FiO2 ratio (PFRatio) was calculated whenever there was documentation of either PaO2 or FiO2. From each documentation, we looked back 24 hours for the latest complement documentation (PaO2 for FiO2 and vice versa) to calculate the ratio. If a complement FiO2 could not be found for PaO2, FiO2 was assumed to be 21%. If a complement PaO2 could not be found for PaO2, PaO2 was calculated using the following formula: 100 – Age (years) * 0.3
- Estimated glomerular flow rate (eGFR) was calculated according to the MDRD study equation: 175 * Creatinine^-1.154^ * Age^-0.203^ * ((Gender == Female)*.742)) * ((Race==Black)*1.212)
- Blood urea nitrogen – creatinine ratio (BUNCr ratio) was calculated whenever there was a blood urea nitrogen documentation and creatinine documentation within a one-hour window as blood urea nitrogen / creatinine. Time of documentation was set as the later of the two.
- Shock index (SI) was calculated whenever there was a heart rate documentation and a systolic blood pressure documentation within a one-hour window as heart rate / systolic blood pressure. Time of documentation was set as the later of the two.

All numeric features were standardized (zero-mean and unit-variance) based on the distribution of the features in the training dataset. Time series data was summarized across various lookback time windows (3h, 6h, 12h, 24h, 48h, 96h) through the following aggregation functions: minimum, maximum, mean, skew, median, count, standard deviation, and last. No binning was performed. No boolean flag for presence/absence was generated.

# eTable 2. Data availability

|  | **Total** | | **Sepsis** | | **Non-sepsis** | |
| --- | --- | --- | --- | --- | --- | --- |
| **Labs and Vital Signs** | **% missing** | **# recorded** | **% missing** | **# recorded** | **% missing** | **# recorded** |
| **ALP** | 25.77 | 1 (0 - 2) | 7.52 | 3 (2 - 7) | 26.36 | 1 (0 - 2) |
| **ALT** | 25.77 | 1 (0 - 2) | 7.48 | 3 (2 - 7) | 26.36 | 1 (0 - 2) |
| **AST** | 26.21 | 1 (0 - 2) | 7.75 | 3 (2 - 7) | 26.81 | 1 (0 - 2) |
| **A-a Gradient** | 97.71 | 0 (0 - 0) | 78.92 | 0 (0 - 0) | 98.32 | 0 (0 - 0) |
| **Albumin** | 25.69 | 1 (0 - 2) | 7.43 | 3 (2 - 7) | 26.29 | 1 (0 - 2) |
| **Anion Gap** | 0 | 4 (3 - 7) | 0 | 14.5 (9 - 22) | 0 | 4 (3 - 7) |
| **BUN** | 0 | 4 (3 - 7) | 0 | 15 (9 - 22) | 0 | 4 (3 - 7) |
| **BUN-Cr ratio** | 0 | 4 (3 - 7) | 0 | 15 (9 - 22) | 0 | 4 (3 - 7) |
| **Base Excess** | 93.21 | 0 (0 - 0) | 64.01 | 0 (0 - 1) | 94.16 | 0 (0 - 0) |
| **Basophils** | 5.15 | 3 (1 - 5) | 2.27 | 8 (4 - 13) | 5.25 | 3 (1 - 5) |
| **Basophils abs** | 6.82 | 3 (1 - 5) | 6.17 | 7 (4 - 13) | 6.84 | 3 (1 - 4) |
| **Bicarbonate** | 0 | 4 (3 - 7) | 0 | 17 (10 - 25) | 0 | 4 (3 - 7) |
| **Bilirubin** | 25.71 | 1 (0 - 2) | 7.43 | 3 (2 - 7) | 26.3 | 1 (0 - 2) |
| **Bilirubin direct** | 67.81 | 0 (0 - 1) | 54.35 | 0 (0 - 1) | 68.25 | 0 (0 - 1) |
| **Calcium** | 0 | 4 (3 - 7) | 0 | 15 (9 - 22) | 0 | 4 (3 - 7) |
| **Calcium ionized** | 89.88 | 0 (0 - 0) | 56.26 | 0 (0 - 3) | 90.97 | 0 (0 - 0) |
| **Chloride** | 0 | 4 (3 - 7) | 0 | 15 (9 - 22) | 0 | 4 (3 - 7) |
| **Cholesterol** | 64.71 | 0 (0 - 1) | 57.62 | 0 (0 - 1) | 64.94 | 0 (0 - 1) |
| **Coombs** | 48.09 | 1 (0 - 1) | 15.78 | 2 (1 - 3) | 49.14 | 1 (0 - 1) |
| **Creatinine** | 0 | 4 (3 - 7) | 0 | 15 (9 - 22) | 0 | 4 (3 - 7) |
| **DBP** | 0 | 24 (14 - 50) | 0 | 141 (79 - 222) | 0 | 24 (14 - 46) |
| **Eosinophils** | 5.04 | 3 (1 - 5) | 2.4 | 8 (4 - 13) | 5.13 | 3 (1 - 5) |
| **Eosinophils abs** | 6.84 | 3 (1 - 5) | 6.3 | 7 (4 - 13) | 6.85 | 3 (1 - 5) |
| **FiO2** | 61.87 | 0 (0 - 8) | 14.91 | 41 (10 - 100) | 63.4 | 0 (0 - 6) |
| **Glucose** | 0.02 | 7 (3 - 25) | 0 | 39 (16 - 101) | 0.02 | 7 (3 - 23) |
| **HCT** | 0 | 4 (3 - 7) | 0 | 16 (9 - 24) | 0 | 4 (3 - 7) |
| **HDL** | 64.62 | 0 (0 - 1) | 57.12 | 0 (0 - 1) | 64.86 | 0 (0 - 1) |
| **HGB** | 0 | 4 (3 - 7) | 0 | 15 (9 - 22) | 0 | 4 (3 - 7) |
| **Heart Rate** | 0 | 28 (16 - 57) | 0 | 162 (95 - 261.75) | 0 | 27 (16 - 53) |
| **HgA1C** | 65.89 | 0 (0 - 1) | 67.36 | 0 (0 - 1) | 65.84 | 0 (0 - 1) |
| **INR** | 20.46 | 1 (1 - 3) | 4.03 | 4 (2 - 8) | 20.99 | 1 (1 - 3) |
| **Immature Granulocyte** | 62.27 | 0 (0 - 2) | 57.62 | 0 (0 - 5) | 62.42 | 0 (0 - 1) |
| **Immature Granulocyte abs** | 60.95 | 0 (0 - 2) | 55.53 | 0 (0 - 6) | 61.13 | 0 (0 - 2) |
| **LDH** | 84.39 | 0 (0 - 0) | 65.82 | 0 (0 - 1) | 84.99 | 0 (0 - 0) |
| **LDL** | 65.65 | 0 (0 - 1) | 58.93 | 0 (0 - 1) | 65.87 | 0 (0 - 1) |
| **Lactic Acid** | 80.86 | 0 (0 - 0) | 45.83 | 1 (0 - 3) | 82 | 0 (0 - 0) |
| **Lipase** | 84.06 | 0 (0 - 0) | 83.41 | 0 (0 - 0) | 84.08 | 0 (0 - 0) |
| **Lymphocytes** | 4.29 | 3 (2 - 5) | 1.9 | 9 (5 - 15) | 4.37 | 3 (1 - 5) |
| **Lymphocytes abs** | 6.48 | 3 (1 - 5) | 6.07 | 8 (4 - 13) | 6.49 | 3 (1 - 5) |
| **MAP** | 0 | 24 (14 - 50) | 0 | 141 (79 - 222) | 0 | 24 (14 - 46) |
| **MCH** | 0 | 4 (3 - 7) | 0 | 15 (9 - 22) | 0 | 4 (3 - 7) |
| **MCHC** | 0 | 4 (3 - 7) | 0 | 15 (9 - 22) | 0 | 4 (3 - 7) |
| **MCV** | 0 | 4 (3 - 7) | 0 | 15 (9 - 22) | 0 | 4 (3 - 7) |
| **MPV** | 0.53 | 4 (3 - 7) | 0.41 | 14 (9 - 22) | 0.53 | 4 (2 - 7) |
| **Magnesium** | 32.26 | 1 (0 - 3) | 7.21 | 7 (3 - 14) | 33.07 | 1 (0 - 3) |
| **Monocytes** | 4.31 | 3 (2 - 5) | 1.9 | 9 (5 - 14) | 4.39 | 3 (1 - 5) |
| **Monocytes abs** | 6.45 | 3 (1 - 5) | 6.21 | 8 (4 - 13) | 6.46 | 3 (1 - 5) |
| **Neutrophils** | 4.29 | 3 (2 - 5) | 1.9 | 9 (5 - 15) | 4.37 | 3 (1 - 5) |
| **Neutrophils abs** | 6.43 | 3 (1 - 5) | 5.98 | 8 (4 - 13) | 6.44 | 3 (1 - 5) |
| **O2 Flow** | 62.2 | 0 (0 - 8) | 15.19 | 38 (8 - 94) | 63.73 | 0 (0 - 6) |
| **PCO2** | 87.95 | 0 (0 - 0) | 37.76 | 1 (0 - 6) | 89.58 | 0 (0 - 0) |
| **P-F Ratio** | 60.44 | 0 (0 - 8) | 12.92 | 45 (11 - 107) | 61.99 | 0 (0 - 7) |
| **PLT** | 0.01 | 4 (3 - 7) | 0 | 15 (9 - 22) | 0.01 | 4 (2 - 7) |
| **PO2** | 87.95 | 0 (0 - 0) | 37.76 | 1 (0 - 6) | 89.58 | 0 (0 - 0) |
| **PT** | 20.46 | 1 (1 - 3) | 4.03 | 4 (2 - 8) | 20.99 | 1 (1 - 3) |
| **PTT** | 27.15 | 1 (0 - 2) | 7.43 | 4 (2 - 8) | 27.79 | 1 (0 - 2) |
| **Phosphorus** | 52.89 | 0 (0 - 2) | 15.14 | 5 (1 - 11) | 54.11 | 0 (0 - 2) |
| **Plasma Protein** | 25.74 | 1 (0 - 2) | 7.57 | 3 (2 - 7) | 26.33 | 1 (0 - 2) |
| **Potassium** | 0.17 | 4 (3 - 7) | 0 | 16 (10 - 24) | 0.18 | 4 (3 - 7) |
| **RBC** | 0 | 4 (3 - 7) | 0 | 15 (9 - 22) | 0 | 4 (3 - 7) |
| **RDW CV** | 0.02 | 4 (3 - 7) | 0 | 15 (9 - 22) | 0.02 | 4 (2 - 7) |
| **RDW FL** | 57.41 | 0 (0 - 4) | 53.67 | 0 (0 - 13) | 57.53 | 0 (0 - 3) |
| **Respiratory Rate** | 0 | 23 (13.25 - 48) | 0 | 147 (82 - 240) | 0 | 22 (13 - 45) |
| **SBP** | 0 | 24 (14 - 50) | 0 | 141 (79 - 222) | 0 | 24 (14 - 46) |
| **Shock Index** | 0 | 25 (15 - 53) | 0 | 150 (86 - 242) | 0 | 24 (14 - 49) |
| **Sodium** | 0 | 4 (3 - 7) | 0 | 16 (9 - 23) | 0 | 4 (3 - 7) |
| **SpO2** | 0 | 23 (13 - 48) | 0 | 146 (80.25 - 235.75) | 0 | 22 (13 - 45) |
| **TSH** | 75.19 | 0 (0 - 0) | 68 | 0 (0 - 1) | 75.43 | 0 (0 - 0) |
| **Temperature** | 0 | 19 (12 - 37) | 0 | 88 (50 - 142) | 0 | 18 (12 - 34) |
| **Triglycerides** | 64.73 | 0 (0 - 1) | 57.34 | 0 (0 - 1) | 64.97 | 0 (0 - 1) |
| **Troponin I** | 53.82 | 0 (0 - 2) | 38.94 | 1 (0 - 3) | 54.31 | 0 (0 - 2) |
| **Urinalysis Ketones** | 94.18 | 0 (0 - 0) | 91.61 | 0 (0 - 0) | 94.26 | 0 (0 - 0) |
| **Urinalysis Leukocyte Esterase** | 82.97 | 0 (0 - 0) | 57.21 | 0 (0 - 1) | 83.81 | 0 (0 - 0) |
| **Urinalysis Nitrite** | 47.98 | 1 (0 - 1) | 18.59 | 1 (1 - 2) | 48.94 | 1 (0 - 1) |
| **Urinalysis SpecificGravity** | 47.98 | 1 (0 - 1) | 18.59 | 1 (1 - 2) | 48.94 | 1 (0 - 1) |
| **Urinalysis pH** | 47.99 | 1 (0 - 1) | 18.59 | 1 (1 - 2) | 48.94 | 1 (0 - 1) |
| **WBC** | 0 | 4 (3 - 7) | 0 | 15 (9 - 22) | 0 | 4 (3 - 7) |
| **eGFR** | 0 | 4 (3 - 7) | 0 | 15 (9 - 22) | 0 | 4 (3 - 7) |
| **pH** | 87.95 | 0 (0 - 0) | 37.76 | 1 (0 - 6) | 89.58 | 0 (0 - 0) |

Percentage of encounters missing labs and vital signs measurements, and number of labs and vital signs measurements per encounter (median and IQR) stratified by sepsis.

Abbreviations: ALP, alkaline phosphatase; ALT, alanine aminotransferase; AST, aspartate aminotransferase; BUN, blood urea nitrogen; Cr, creatinine; DBP, diastolic blood pressure; HCT, hematocrit; HDL, high density lipoprotein; HGB, hemoglobin: INR, international normalized ratio; LDH, lactate dehydrogenase; LDL, low density lipoprotein; MAP, mean arterial pressure; MCH, mean corpuscular hemoglobin; MCHC, mean corpuscular hemoglobin concentration; MCV, mean corpuscular volume; MPV, mean platelet volume; P-F, PaO2 to FiO2 ratio; PCO2, partial pressure of carbon dioxide; PLT, platelets; PO2, partial pressure of oxygen; PT, prothrombin time; PTT, partial thromboplastin time; RBC, red blood cell count; RDW CV, red cell distribution width coefficient of variation; RDW FL, red cell distribution width femtoliters; SBP, systolic blood pressure; TSH, thyroid stimulating hormone; WBC, white blood cell count; eGFR, estimated glomerular filtration rate.

# eMethods 3. Index time identification for sepsis and non-sepsis cohorts

For each patient encounter, a single index time was identified, and prediction was performed six hours prior to that index time.

For non-sepsis patients, index time was the maximum of [12 hours into admission] or [mid point between admission and discharge]:


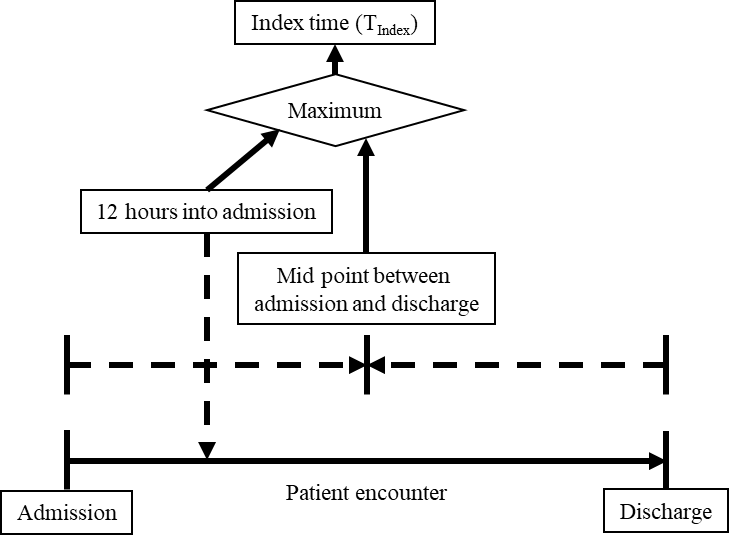


For sepsis patients, based on the elements that qualified the patient for meeting sepsis criteria, the time of suspicion of infection was also the time of sepsis and the time of index:


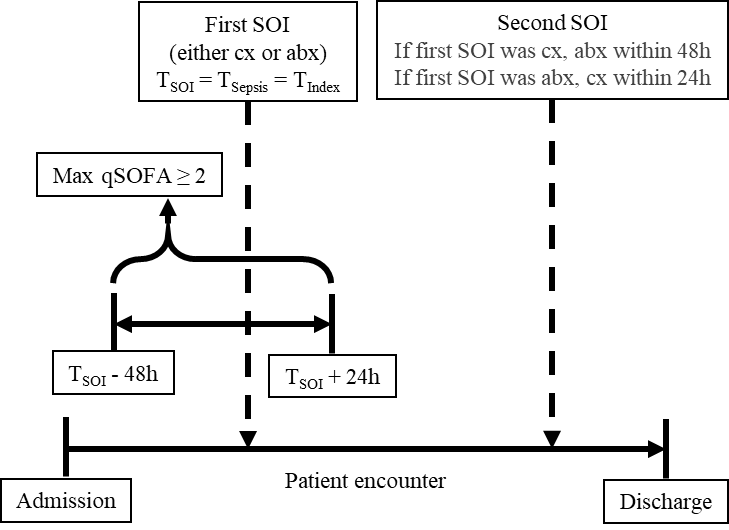


Abbreviations: SOI, suspicion of infection; qSOFA, quick sequential organ failure assessment; cx, cultures; abx, antibiotics.

# eFigure 1. Cohort selection PRISMA-style diagram


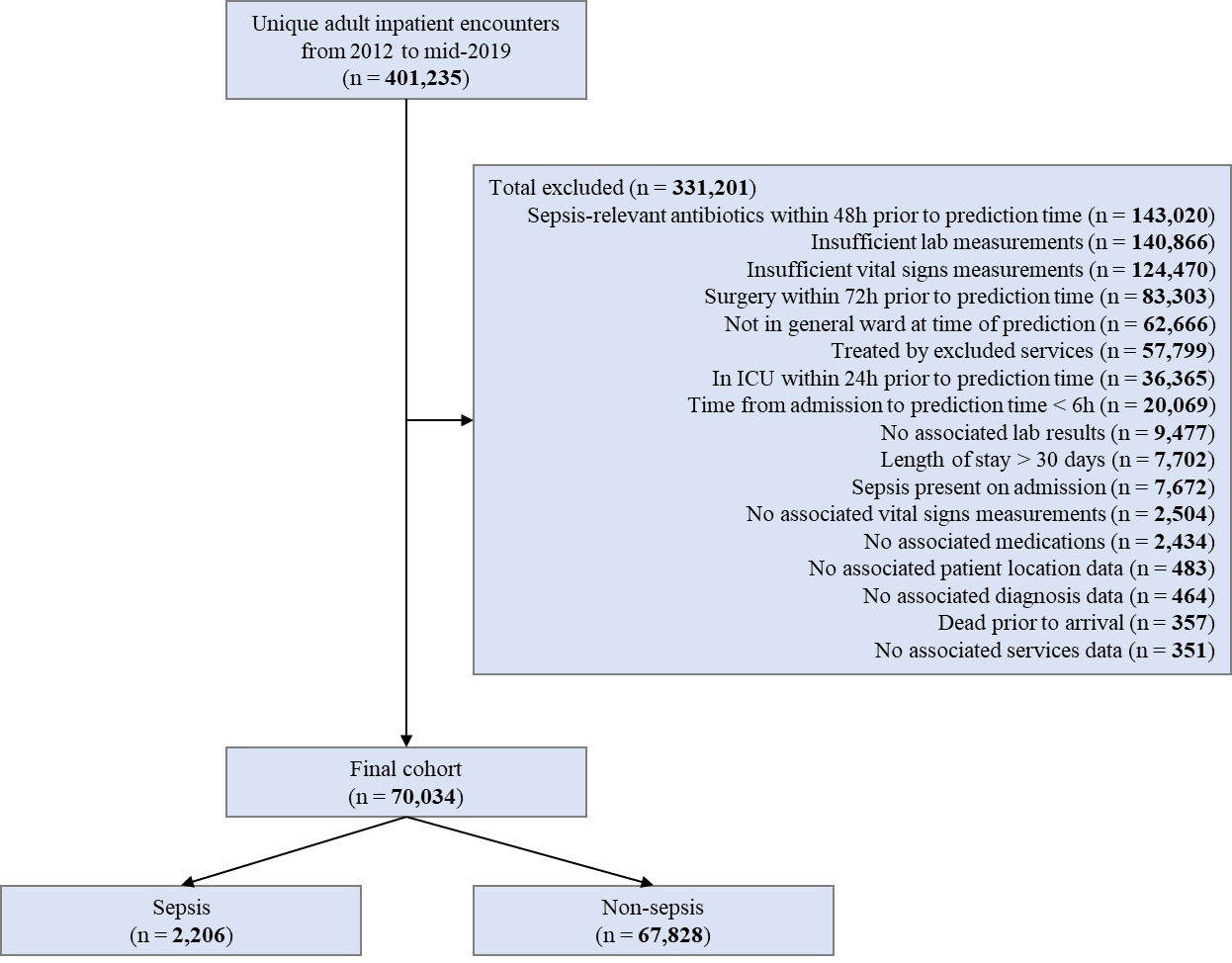


Each encounter can meet multiple exclusion criteria, thus the sum of number of encounters excluded by each criteria is greater than the total number of excluded encounters.

# eTable 3. Cohort characteristics: comorbidities

| **Variable** | **Total** (n = 70,034) | **Sepsis** (n = 2,206) | **Non-sepsis** (n = 67,828) | **p**^a^ | |
| --- | --- | --- | --- | --- | --- |
| **AIDS/HIV, n (%)** | 540 (0.8%) | 11 (0.5%) | 529 (0.8%) | 0.173 |  |
| **Alcohol abuse, n (%)** | 1,120 (1.6%) | 37 (1.7%) | 1,083 (1.6%) | 0.833 |  |
| **Blood loss anemia, n (%)** | 917 (1.3%) | 31 (1.4%) | 886 (1.3%) | 0.759 |  |
| **Cardiac arrhythmias, n (%)** | 20,191 (28.8%) | 1,142 (51.8%) | 19,049 (28.1%) | < 0.01 | ***** |
| **Chronic pulmonary disease, n (%)** | 17,823 (25.4%) | 782 (35.4%) | 17,041 (25.1%) | < 0.01 | ***** |
| **Coagulopathy, n (%)** | 5,679 (8.1%) | 505 (22.9%) | 5,174 (7.6%) | < 0.01 | ***** |
| **Congestive heart failure, n (%)** | 19,846 (28.3%) | 908 (41.2%) | 18,938 (27.9%) | < 0.01 | ***** |
| **Deficiency anemia, n (%)** | 3,655 (5.2%) | 132 (6.0%) | 3,523 (5.2%) | 0.111 |  |
| **Depression, n (%)** | 11,881 (17.0%) | 419 (19.0%) | 11,462 (16.9%) | 0.011 |  |
| **Diabetes, complicated, n (%)** | 8,717 (12.4%) | 377 (17.1%) | 8,340 (12.3%) | < 0.01 | ***** |
| **Diabetes, uncomplicated, n (%)** | 13,099 (18.7%) | 327 (14.8%) | 12,772 (18.8%) | < 0.01 | ***** |
| **Drug abuse, n (%)** | 4,434 (6.3%) | 97 (4.4%) | 4,337 (6.4%) | < 0.01 | ***** |
| **Fluid and electrolyte  disorders, n (%)** | 20,911 (29.9%) | 1,300 (58.9%) | 19,611 (28.9%) | < 0.01 | ***** |
| **Hypertension, n (%)** | 31,368 (44.8%) | 1,067 (48.4%) | 30,301 (44.7%) | < 0.01 | ***** |
| **Hypothyroidism, n (%)** | 9,001 (12.9%) | 371 (16.8%) | 8,630 (12.7%) | < 0.01 | ***** |
| **Liver disease, n (%)** | 6,061 (8.7%) | 307 (13.9%) | 5,754 (8.5%) | < 0.01 | ***** |
| **Lymphoma, n (%)** | 2,960 (4.2%) | 124 (5.6%) | 2,836 (4.2%) | < 0.01 | ***** |
| **Metastatic cancer, n (%)** | 6,676 (9.5%) | 261 (11.8%) | 6,415 (9.5%) | < 0.01 | ***** |
| **Obesity, n (%)** | 5,309 (7.6%) | 245 (11.1%) | 5,064 (7.5%) | < 0.01 | ***** |
| **Other neurological disorders, n (%)** | 4,622 (6.6%) | 205 (9.3%) | 4,417 (6.5%) | < 0.01 | ***** |
| **Paralysis, n (%)** | 1,861 (2.7%) | 105 (4.8%) | 1,756 (2.6%) | < 0.01 | ***** |
| **Peptic ulcer disease excluding bleeding, n (%)** | 566 (0.8%) | 25 (1.1%) | 541 (0.8%) | 0.107 |  |
| **Peripheral vascular disorders, n (%)** | 5,514 (7.9%) | 272 (12.3%) | 5,242 (7.7%) | < 0.01 | ***** |
| **Psychoses, n (%)** | 1,243 (1.8%) | 45 (2.0%) | 1,198 (1.8%) | 0.381 |  |
| **Pulmonary circulation disorders, n (%)** | 3,301 (4.7%) | 241 (10.9%) | 3,060 (4.5%) | < 0.01 | ***** |
| **Renal failure, n (%)** | 17,758 (25.4%) | 769 (34.9%) | 16,989 (25.0%) | < 0.01 | ***** |
| **Rheumatoid arthritis/collagen vascular diseases, n (%)** | 2,911 (4.2%) | 111 (5.0%) | 2,800 (4.1%) | 0.042 |  |
| **Solid tumor without metastasis, n (%)** | 9,671 (13.8%) | 384 (17.4%) | 9,287 (13.7%) | < 0.01 | ***** |
| **Valvular disease, n (%)** | 7,699 (11.0%) | 487 (22.1%) | 7,212 (10.6%) | < 0.01 | ***** |
| **Weight loss, n (%)** | 5,987 (8.5%) | 450 (20.4%) | 5,537 (8.2%) | < 0.01 | ***** |

Statistical significance (p < 0.01) indicated by *
Elixhauser comorbidities were coded according to Moore, Med Care, 2017.

# eMethods 4. XGBoost hyperparameter optimization

Random search on the training set was used to optimize XGBoost hyperparameters.

The fixed parameters were as follows:

| **Parameter** | **Value** |
| --- | --- |
| **tree_method** | hist |
| **grow_policy** | depthwise |
| **single_precision_histogram** | True |
| **n_estimators** | 100 |

The parameter spaces for optimization were as follows:

| **Parameter** | **Min** | **Max** | **Distribution** |
| --- | --- | --- | --- |
| **subsample** | 0.2 | 1.0 | Uniform |
| **colsample_bytree** | 0.2 | 1.0 | Uniform |
| **max_depth** | 2 | 32 | Uniform |
| **eta** | 1e-4 | 1 | Log uniform |
| **gamma** | 1e-2 | 1e2 | Log uniform |
| **max_bin** | 4 | 128 | Uniform |
| **min_child_weight** | 1 | 100 | Log uniform |
| **max_delta_step** | 0 | 1000 | Uniform |

At each iteration, for each combination of parameters randomly sampled from the above distribution, 3-fold cross validation was repeated 3 times. Area under precision recall curve (AUPRC) was computed for each of the 9 splits. 300 iterations were performed yielding a total of 2,700 splits. The distribution of mean AUPRCs were as follows:


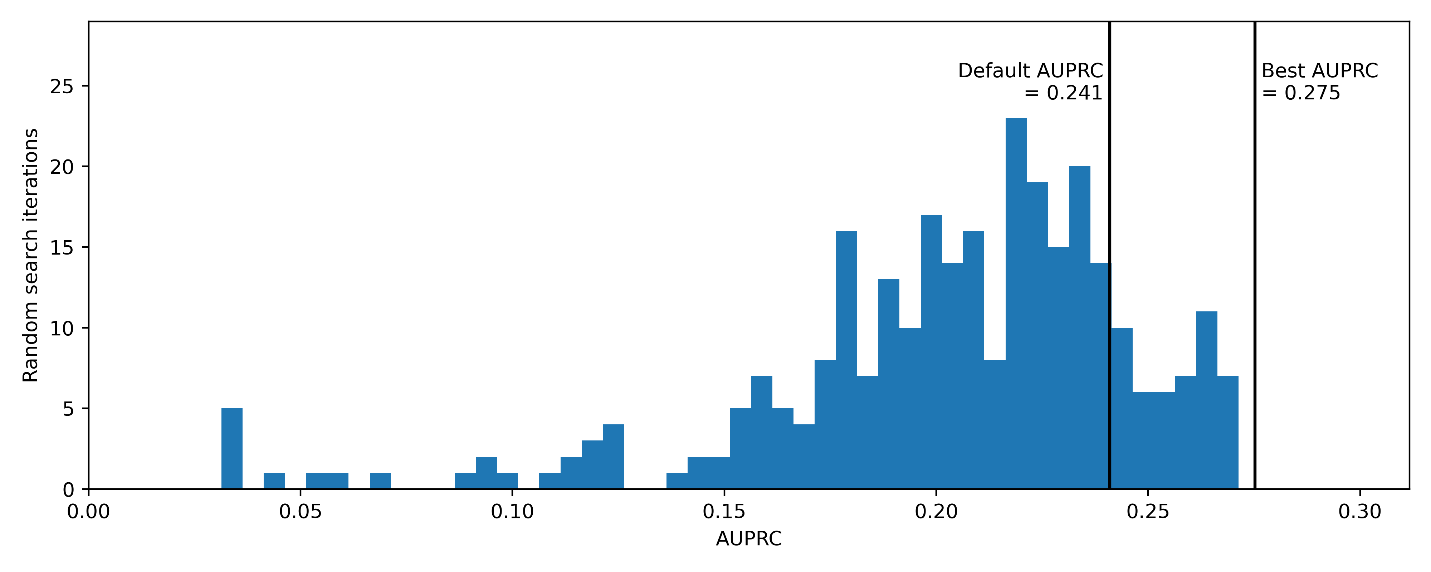


The best mean AUPRC found through random search was 0.275 compared to the median of 0.211 and 0.241 of an unoptimized XGBoost model using default parameters.

For each parameter, the parameter value was plotted against AUPRC and training time. For the AUPRC plots, each point represents a split whereas for the training time plot, each point represents the mean per iteration.


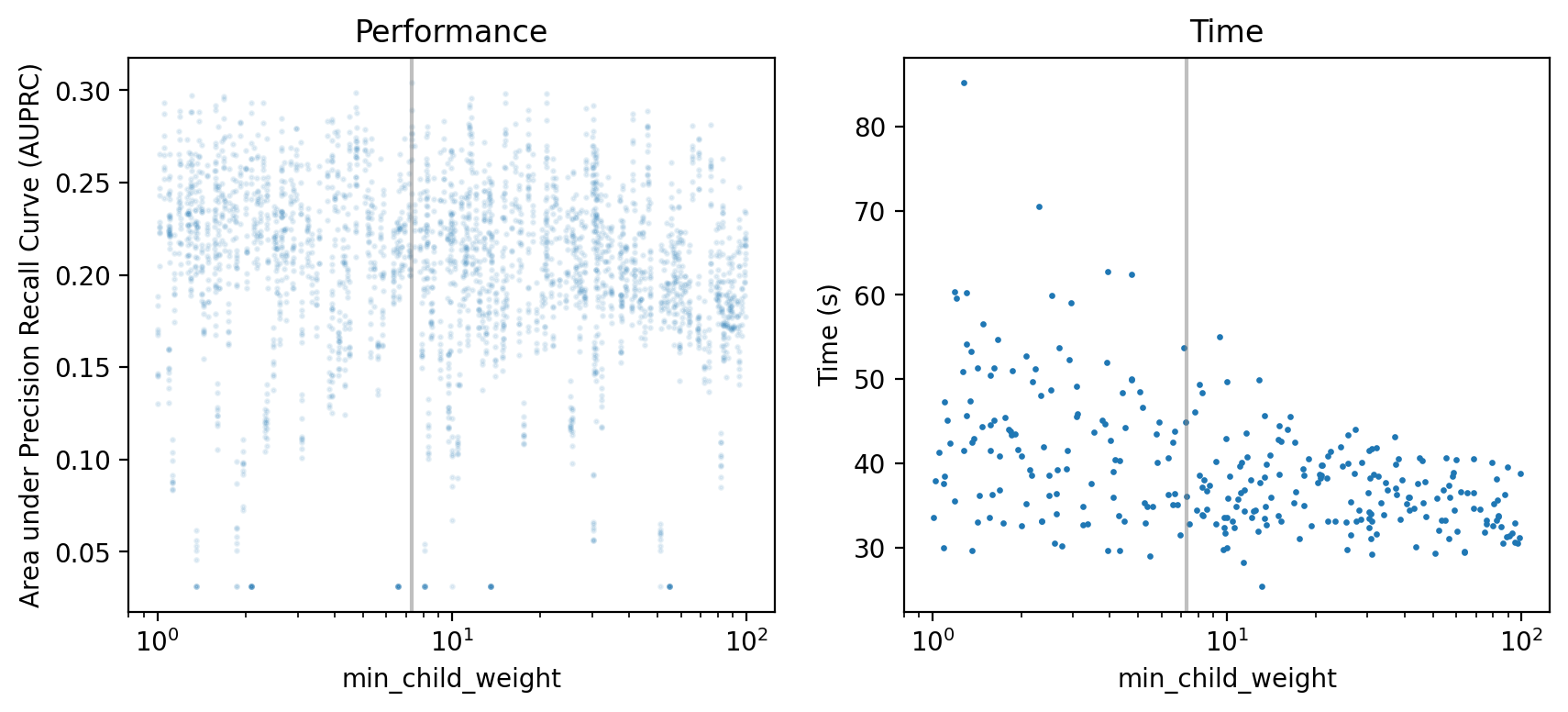

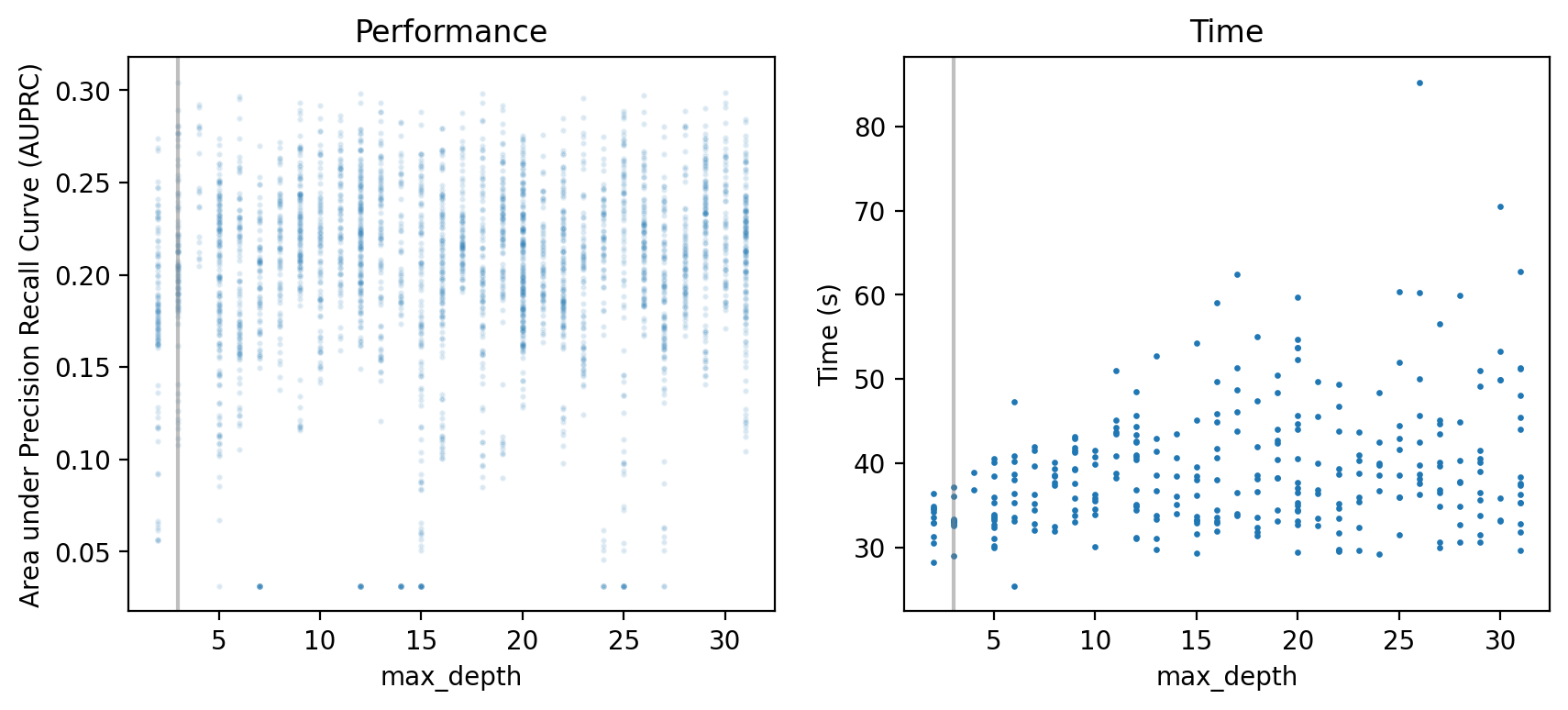

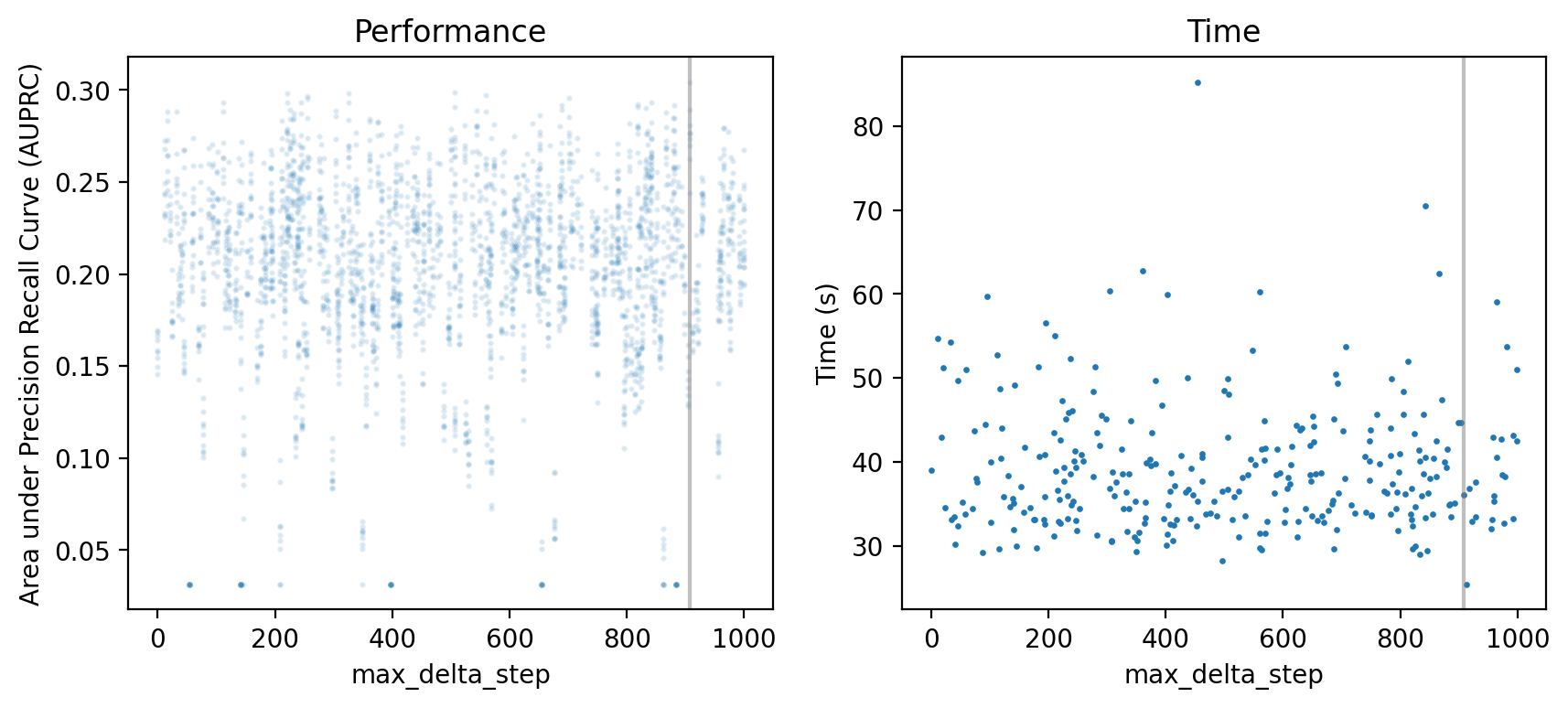

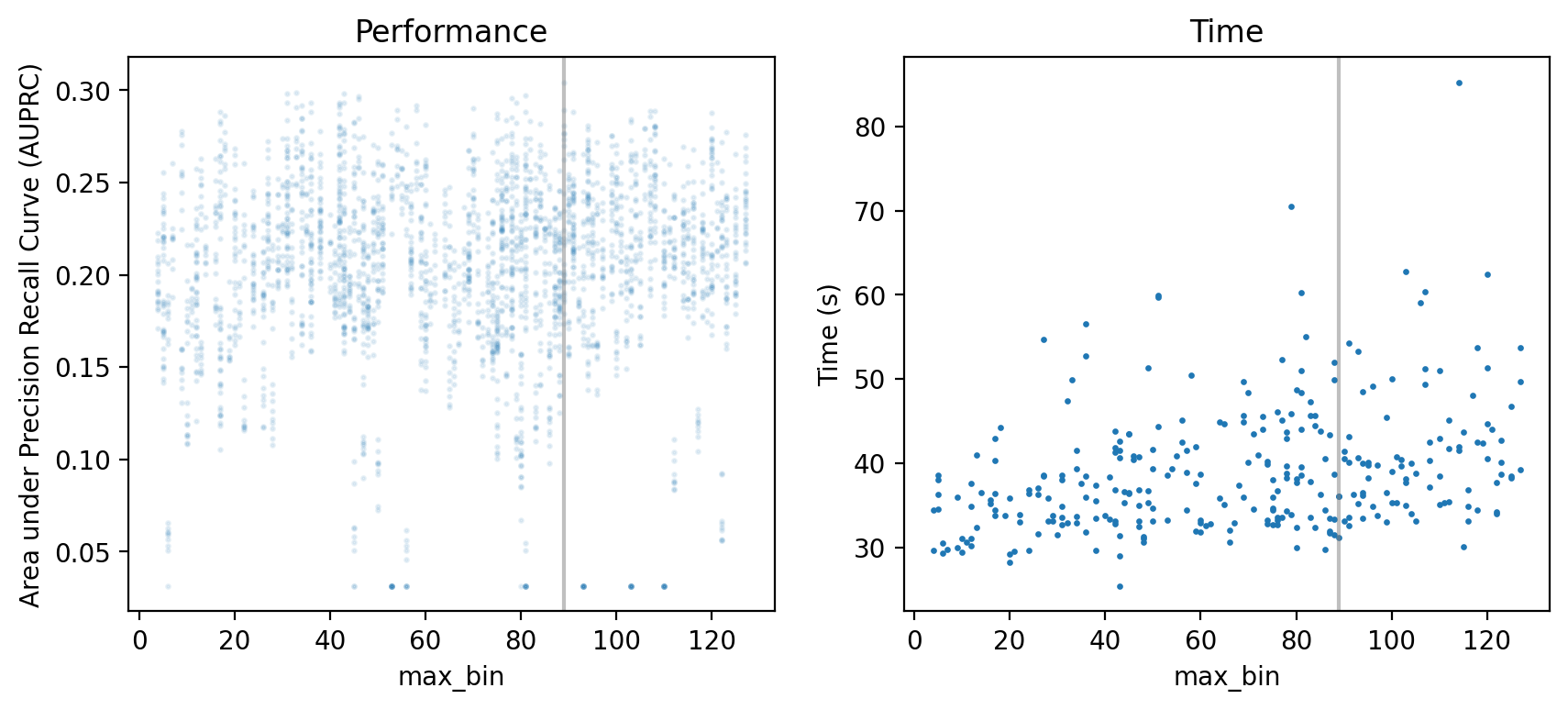

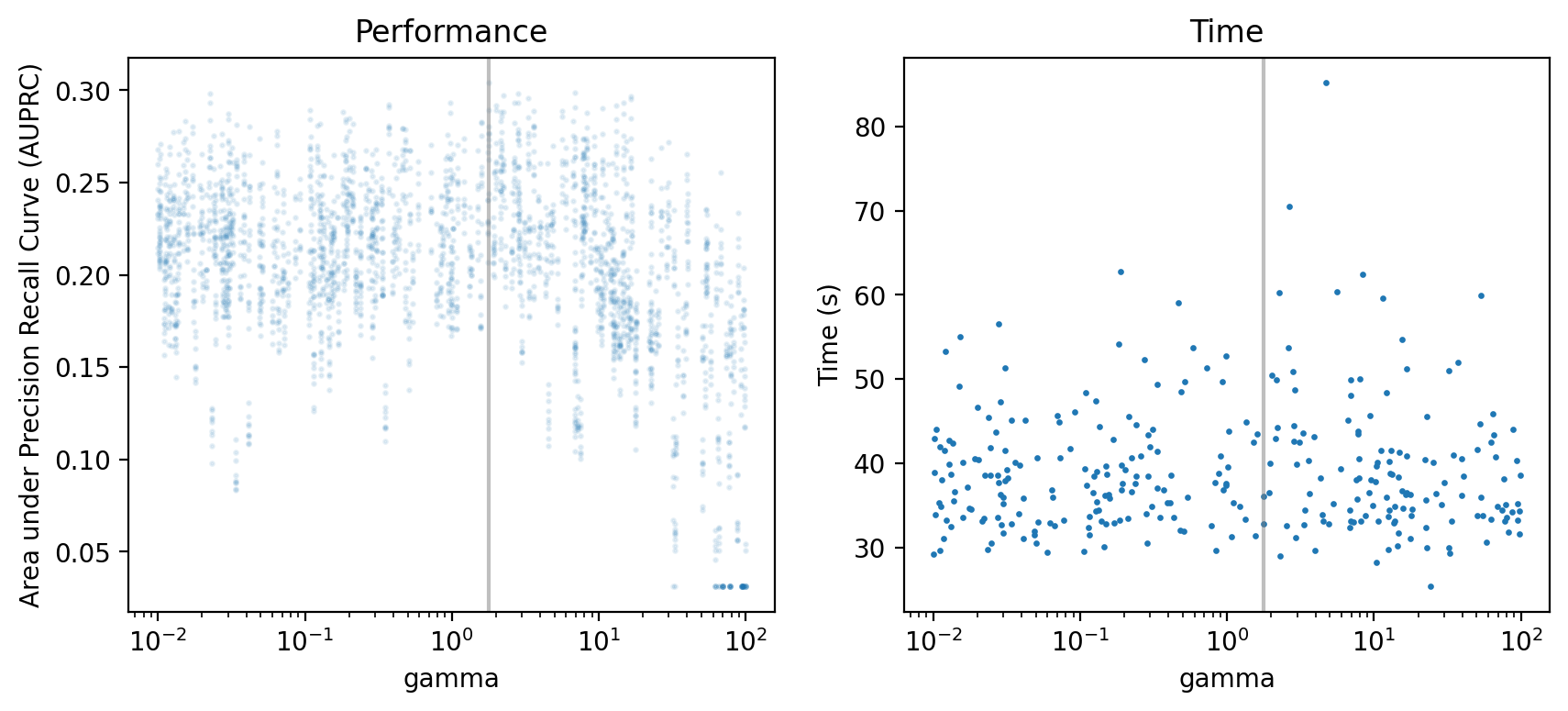

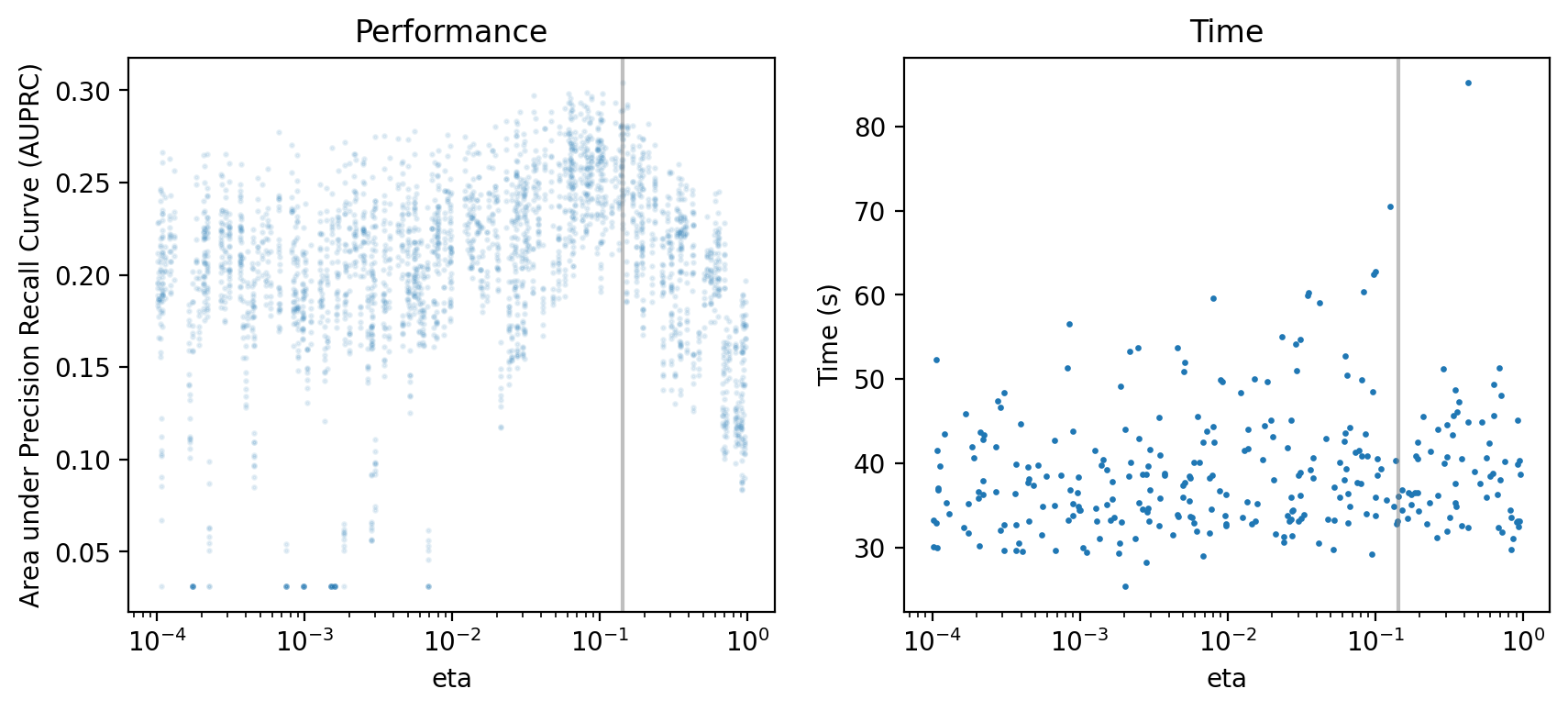

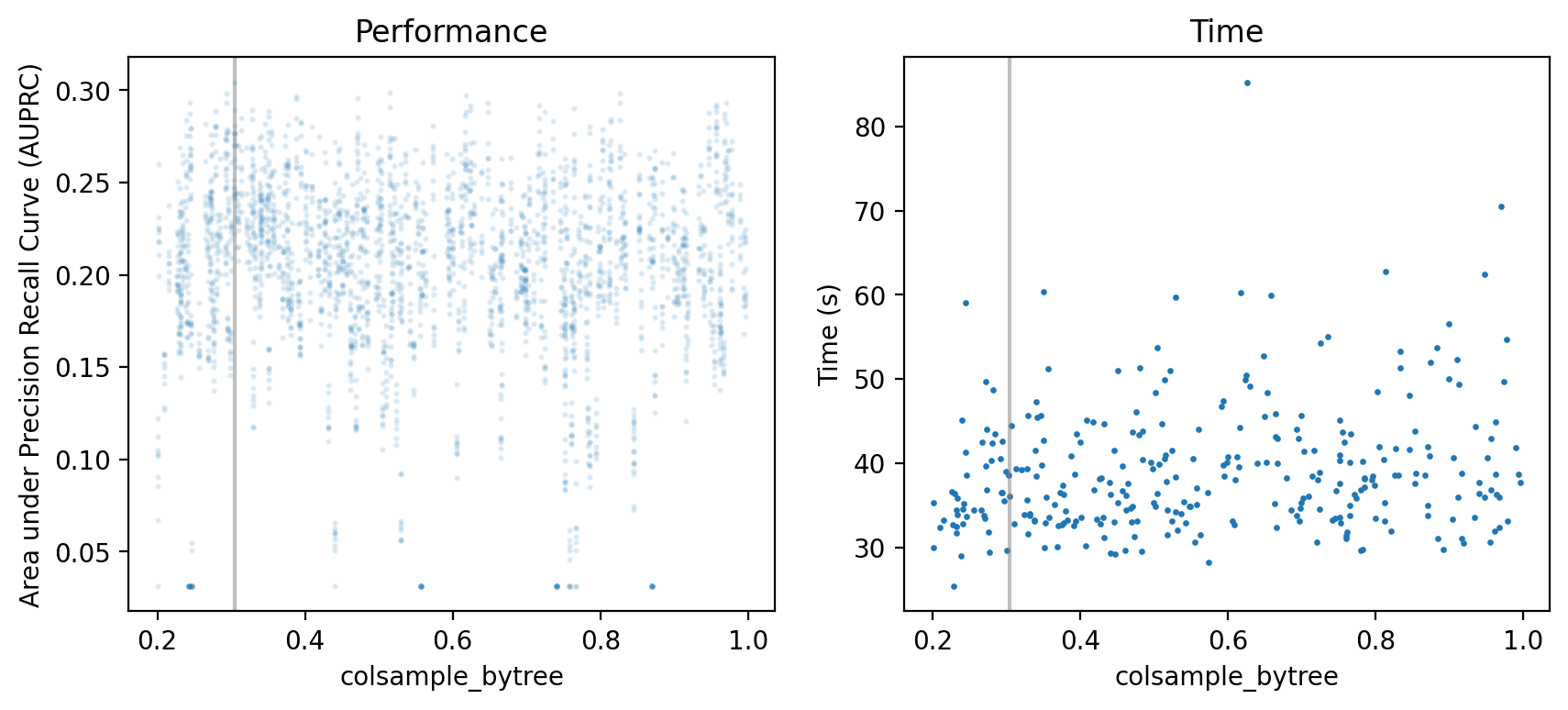

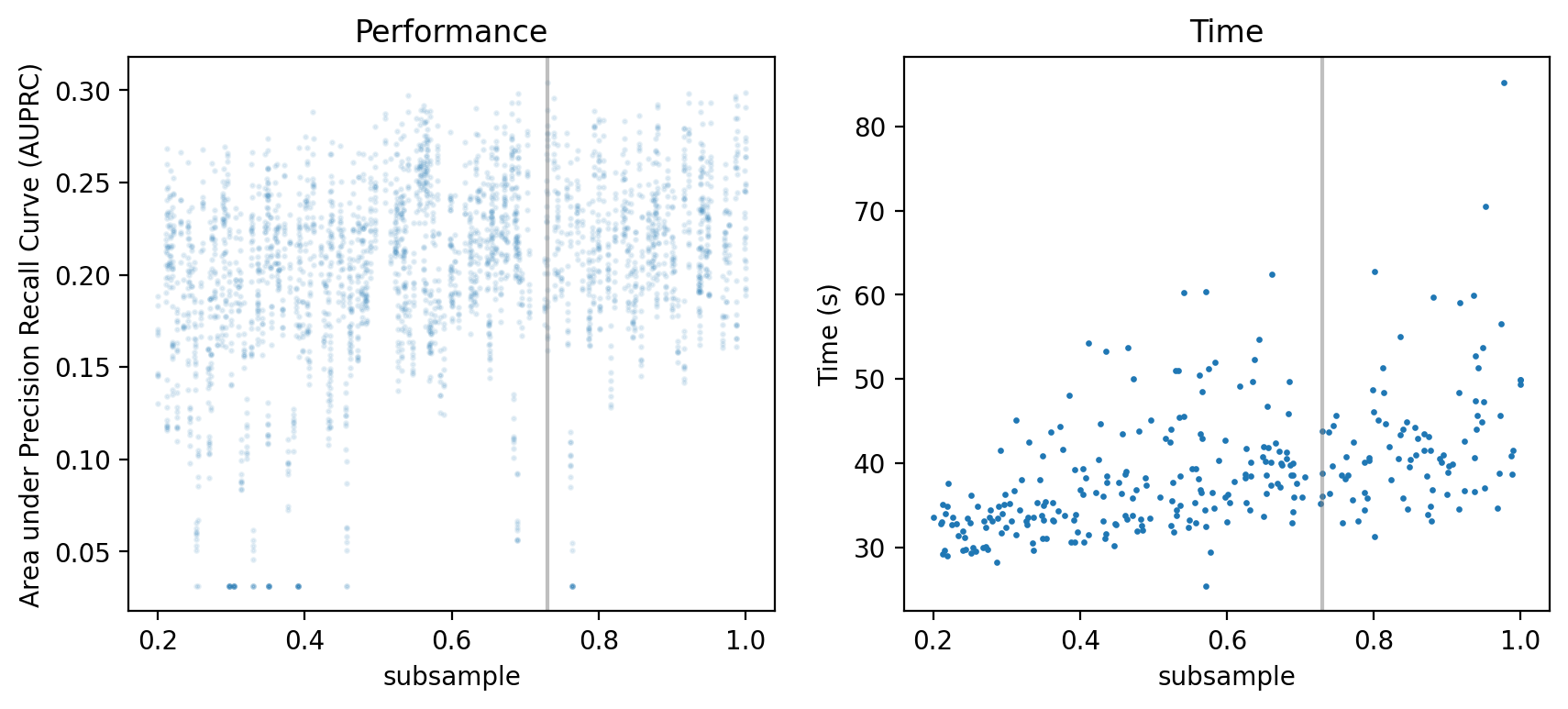


**eTable 4. SHAP values of optimized XGBoost model**

| **Features** | **Absolute SHAP value**  **(median [IQR])** |
| --- | --- |
| **Time to pred time (h)** | 0.262 (0.256 - 0.277) |
| **Age (years)** | 0.125 (0.112 - 0.141) |
| **NEWS2\|Score** | 0.119 (0.097 - 0.132) |
| **qSOFA\|Score** | 0.098 (0.066 - 0.122) |
| **SBP (min 96h)** | 0.097 (0.064 - 0.125) |
| **RespiratoryRate (max 48h)** | 0.086 (0.071 - 0.115) |
| **ShockIndex (max 12h)** | 0.063 (0.050 - 0.069) |
| **Coombs (count 48h)** | 0.063 (0.047 - 0.089) |
| **SBP (min 48h)** | 0.058 (0.047 - 0.080) |
| **ShockIndex (max 24h)** | 0.052 (0.034 - 0.067) |
| **Admitted through ED** | 0.051 (0.030 - 0.066) |
| **MCHC (max 96h)** | 0.049 (0.030 - 0.072) |
| **Temperature (count 96h)** | 0.047 (0.035 - 0.065) |
| **HeartRate (delta 96h)** | 0.047 (0.040 - 0.059) |
| **WBC (median 12h)** | 0.042 (0.035 - 0.046) |
| **RespiratoryRate (max 96h)** | 0.039 (0.022 - 0.054) |
| **Anticonvulsants** | 0.035 (0.022 - 0.048) |
| **RespiratoryRate (max 24h)** | 0.035 (0.025 - 0.055) |
| **Potassium (median 12h)** | 0.033 (0.021 - 0.042) |
| **Temperature (max 12h)** | 0.033 (0.025 - 0.042) |
| **Coombs (median 48h)** | 0.033 (0.027 - 0.054) |
| **Miscellaneous respiratory agents** | 0.032 (0.022 - 0.041) |
| **SBP (min 24h)** | 0.029 (0.015 - 0.057) |
| **SpO2 (std 96h)** | 0.025 (0.013 - 0.045) |
| **BMI** | 0.025 (0.014 - 0.045) |

20 bootstrap samples were generated based on the training set, and for each feature, SHAP values (median and IQR) were calculated across bootstrap samples. The top 25 features based on absolute SHAP values are shown.

Abbreviations: NEWS2, National Early Warning Score 2; qSOFA, quick Sequential Organ Failure Assessment; SBP, systolic blood pressure; MCHC, mean corpuscular hemoglobin concentration; WBC, white blood cell; SpO2, oxygen saturation; BMI, body mass index.

# eFigure 2. Feature selection for lite model


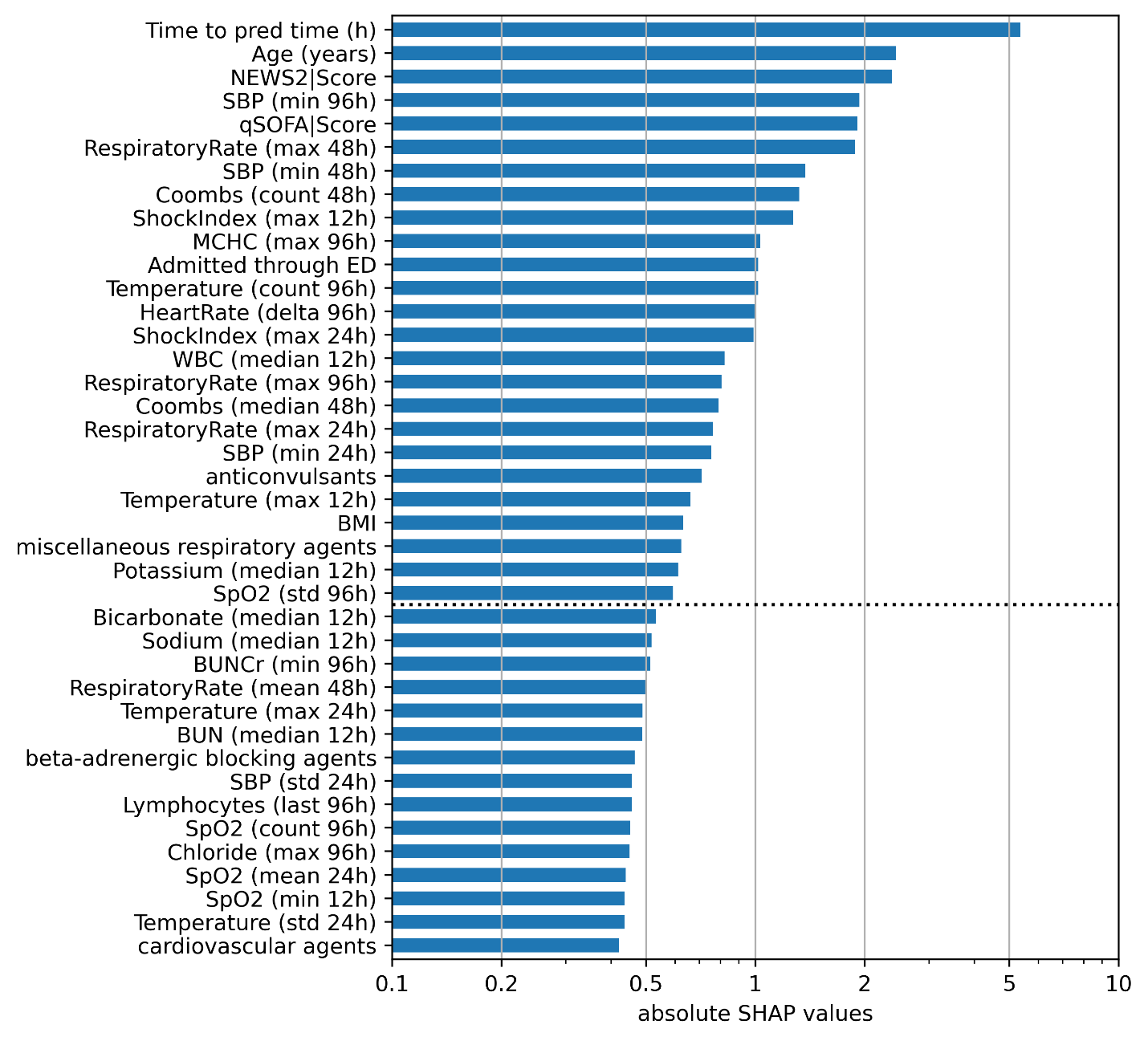


20 bootstrap samples were generated based on the training set, and for each feature, absolute SHAP values were summed across bootstrap samples.

Based on relative drop-off in SHAP value, the cutoff (denoted by the dotted horizontal line) was drawn, and all of the features above the cutoff were used for the “lite” version of the XGBoost model (**XGB lite**).

# eFigure 3. Logistic regression hyperparameter optimization


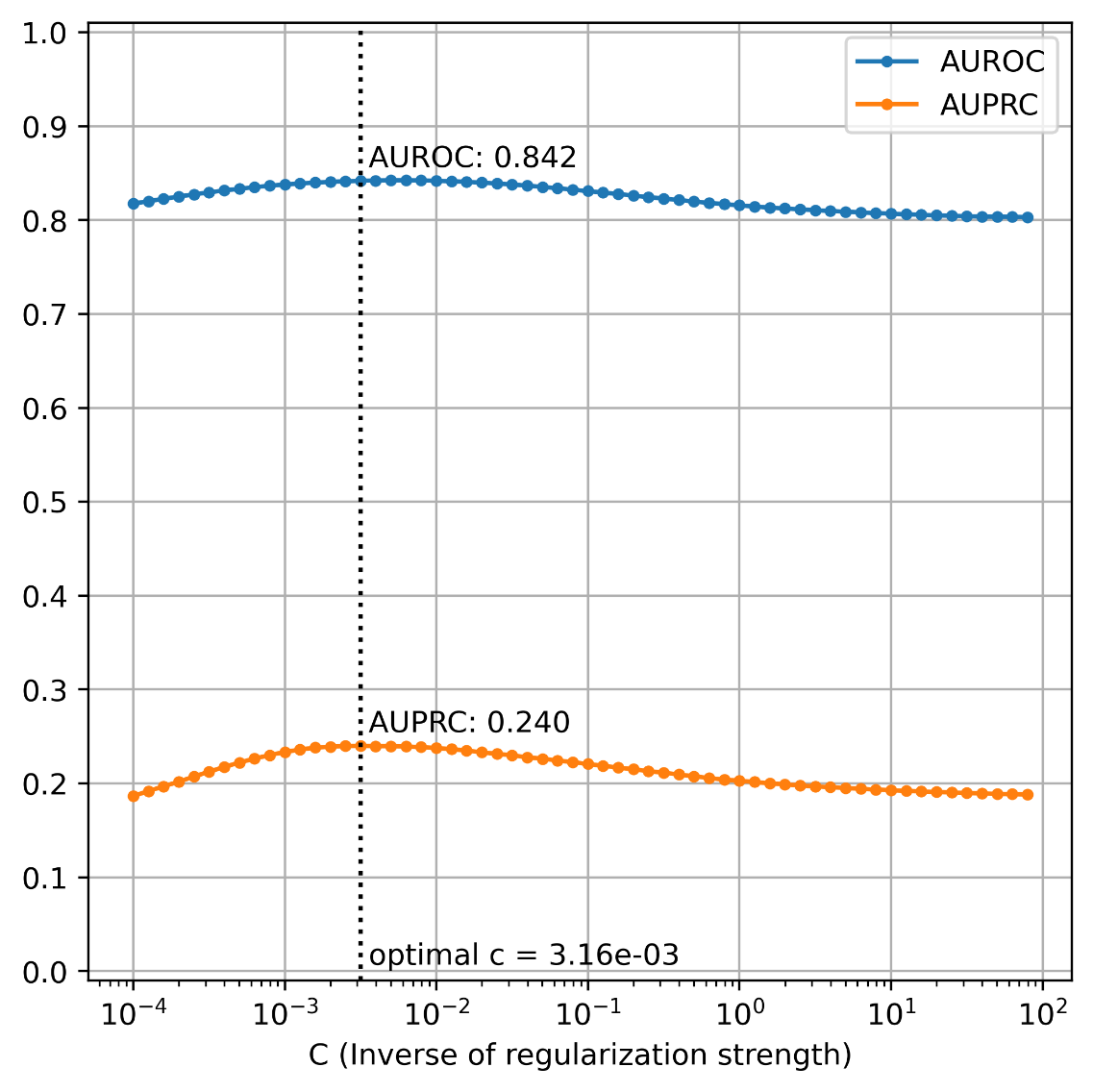


Using grid search with 3-fold, 3-repeat stratified cross validation on the training set, the optimal C (inverse regularization strength) parameter was searched between 10e-4 to 10e2 and was determined to be 3.16e-3, yielding a mean AUPRC of 0.240. This value of C was then used for the logistic regression model (**LogReg**).

# eTable 4. Model performance comparison

| **Model** | **AUROC** | **AUPRC** |
| --- | --- | --- |
| **XGB opt** | **0.862 ± 0.011** | **0.294 ± 0.021** |
| **XGB lite** | 0.856 ± 0.006 | 0.244 ± 0.013 |
| **XGB unopt** | 0.857 ± 0.007 | 0.287 ± 0.017 |
| **LogReg** | 0.857 ± 0.008 | 0.256 ± 0.024 |
| **NEWS2** | 0.699 ± 0.012 | 0.092 ± 0.009 |
| **qSOFA** | 0.705 ± 0.013 | 0.079 ± 0.006 |
| **SIRS** | 0.679 ± 0.010 | 0.066 ± 0.004 |

Model performance distributions were determined through 20 bootstrap samples on the test dataset.

Abbreviations: AUROC, area under receiver operating characteristic curve; AUPRC, area under precision recall curve; XGB opt, optimized XGBoost model; XGB lite, simple XGBoost model; XGB unopt, unoptimized, out-of-the-box XGBoost model; LogReg, logistic regression; NEWS2, National Early Warning Score 2; qSOFA, quick Sequential Organ Failure Assessment; SIRS, Systemic Inflammatory Response Syndrome.

# eFigure 4. Calibration plot for optimized XGBoost model


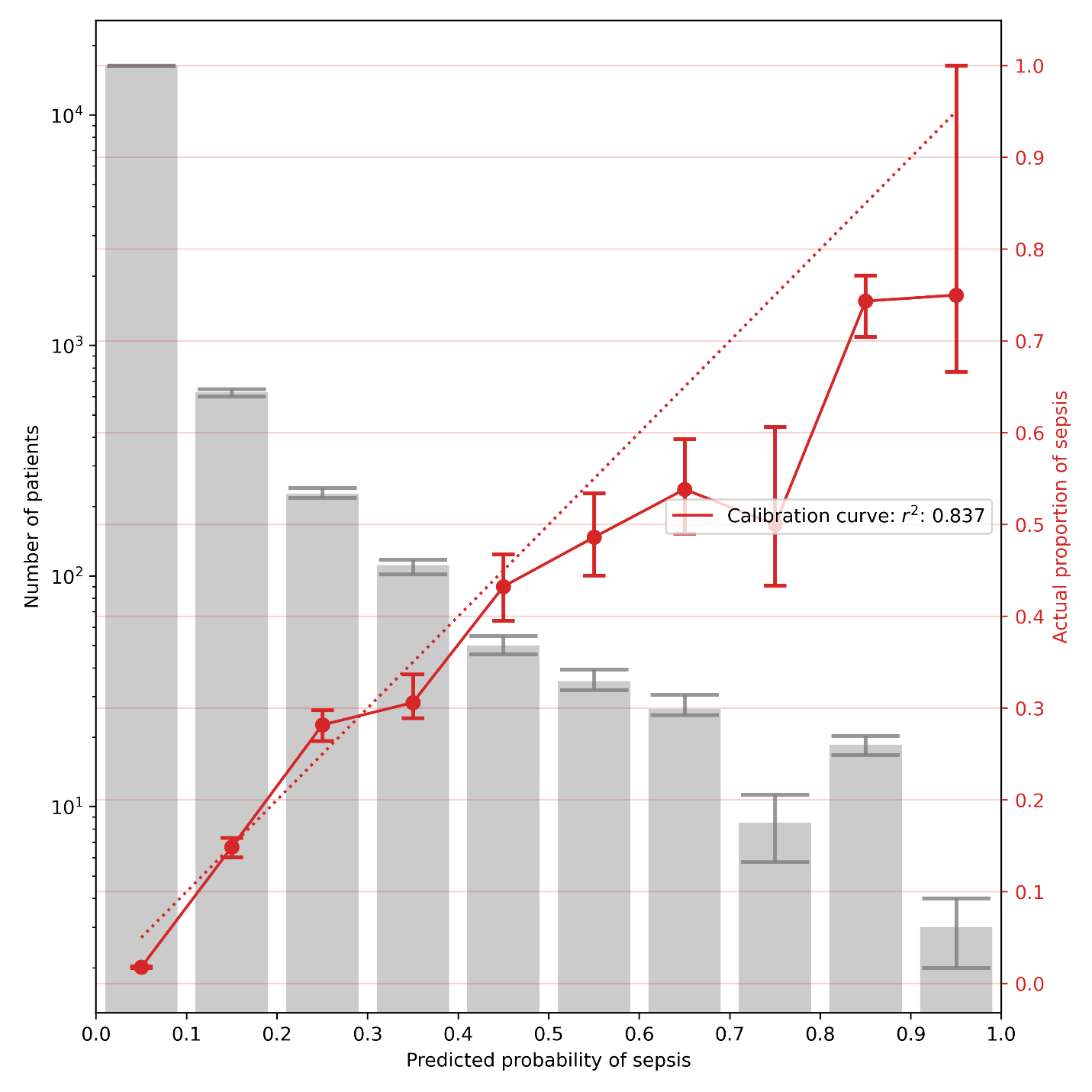


For each of the 20 bootstrap samples on the test set, subjects were binned into deciles of predicted probability of sepsis. The grey bar plot and left y-axis represents the number of subjects in each bin (median and IQR). The red line plot and right y-axis represents the proportion of actual septic subjects in each bin (median and IQR).

# eTable 4. Pseudo-prospective trial, alert confusion matrix

|  | **Sepsis** | **Non-sepsis** |  |
| --- | --- | --- | --- |
| **Alerted** | 388 | 3144 | PPV = 11.0% |
| **Not alerted** | 169 | 13740 |  |
|  | Sensitivity = 69.7% | Specificity = 81.4% | F1 = 19.0% |

Based on the 17,441 encounters in the test dataset, after application of exclusions. Alerts for non-sepsis patients could be from any part of the patient encounter whereas alerts for sepsis patients can only be from before sepsis onset.

# eTable 5. Pseudo-prospective trial, time to intervention or outcome for alerted subjects

| **Intervention or Outcome** | **n (%)** | **Time to event (h), median (IQR)** |
| --- | --- | --- |
| **Sepsis-relevant Cultures** | 1,376 (39.0%) | 31.1 (11.4 - 75.2) |
| **Sepsis-relevant Anti-infectives** | 991 (28.1%) | 52.6 (20.8 - 115.7) |
| **Ventilator Initiation** | 225 (6.4%) | 65.5 (25.7 - 135.3) |
| **Sepsis Onset** | 388 (11.0%) | 29.8 (11.4 - 71.6) |
| **ICU Transfer** | 371 (10.5%) | 57.1 (17.8 - 128.5) |
| **Death** | 164 (4.6%) | 191.5 (81.5 - 320.7) |

# eFigure 5. Pseudoprospective trial, patient trajectory visualizations

Vertical solid blue line represents time of sepsis whereas the dotted blue line represents the first time in the encounter the predicted probability of sepsis crossed the threshold, which would have triggered an alert. Each black tick on the x-axis represents 24 hours whereas each red tick represents 6 hours.

Abbreviations: Cx, sepsis-relevant cultures; Abx, sepsis-relevant anti-infectives; Vent, ventilator; WBC, white blood cell; PLT, platelets; BUN, blood urea nitrogen; DBP, diastolic blood pressure; SBP, systolic blood pressure; MAP, mean arterial pressure; PFRatio, PaO2 FiO2 ratio.

Example of alert success (true positive):


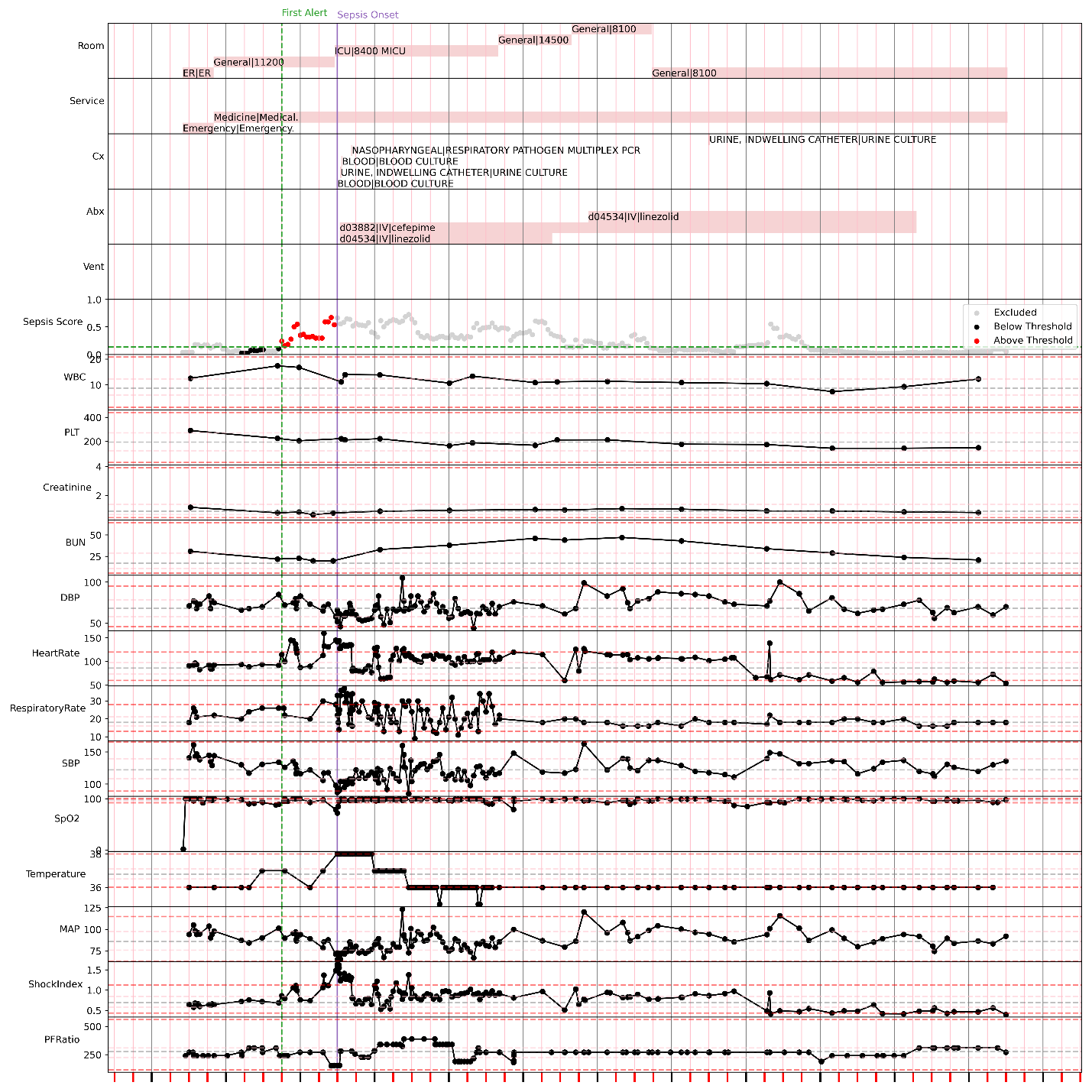


An alert fired roughly 18 hours prior to sepsis-relevant culture collection, sepsis-relevant anti-infective administration, sepsis onset, and ICU transfer.

Example of alert failure (false negative):


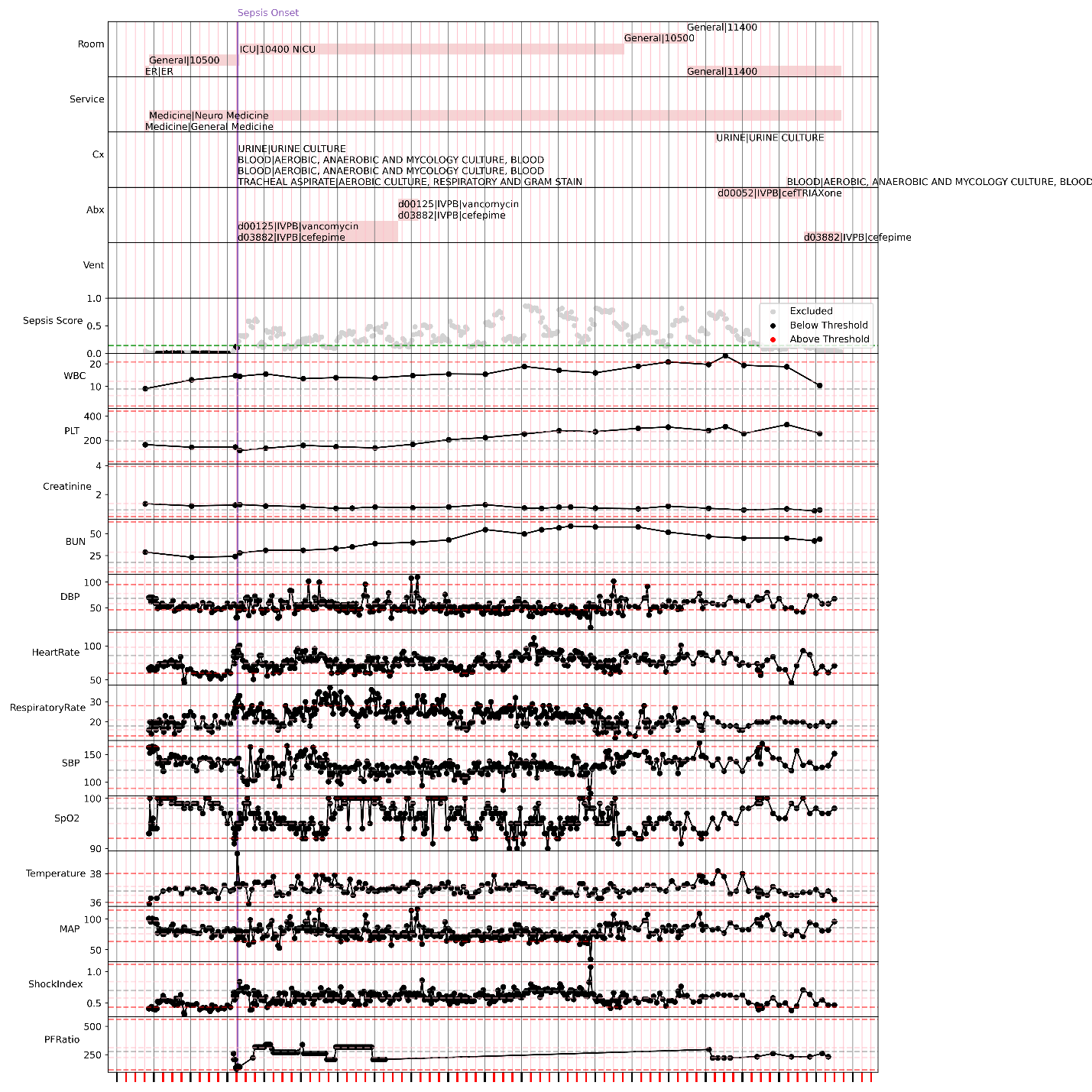


The sepsis risk score was consistently low up to the point of sepsis onset and ICU transfer.

Example of alert failure (false negative):


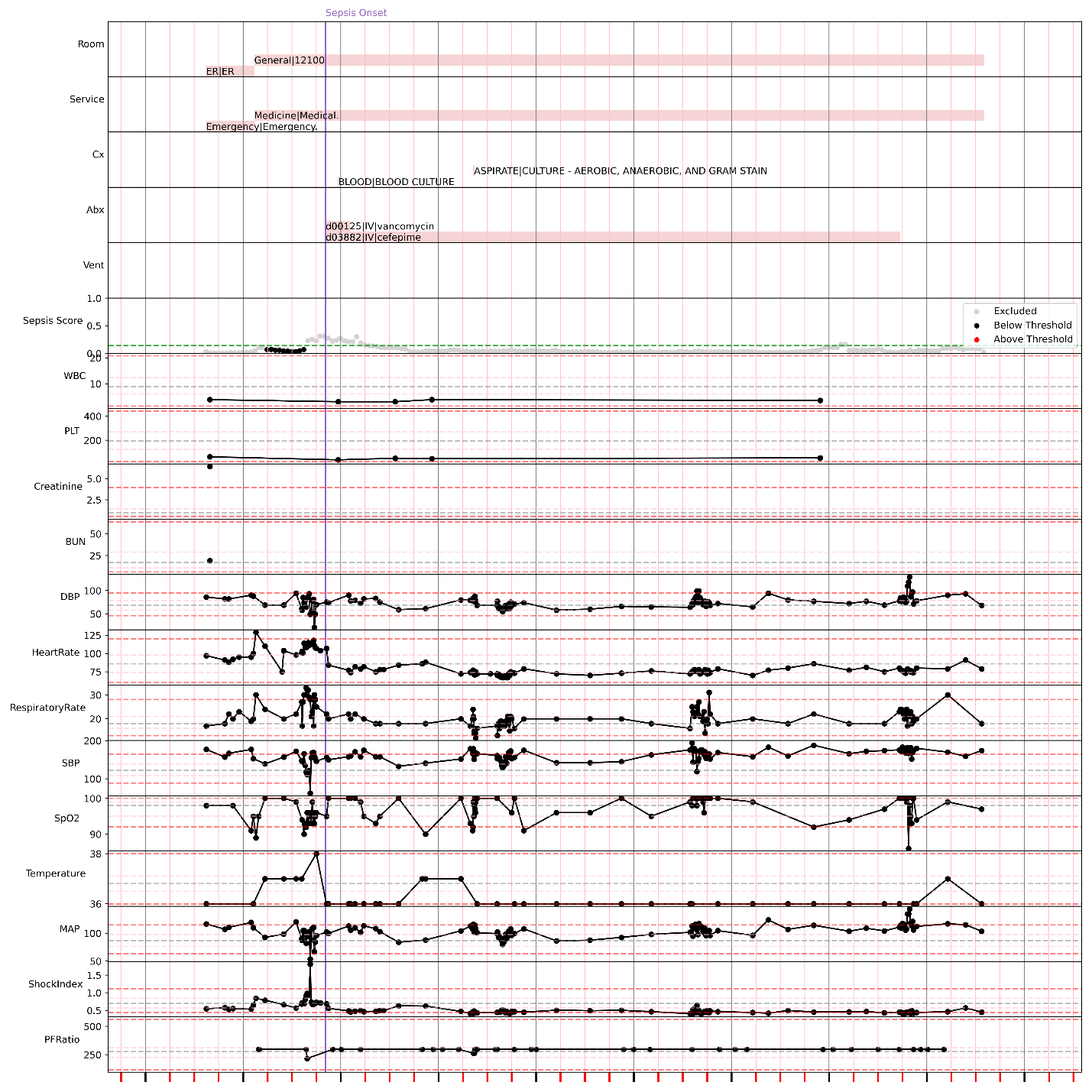


While there were scores crossing the threshold preceding sepsis onset, they were suppressed due to the lack of common labs (CBC/BMP) in the 24 hours preceding evaluation time.

Example of model success (true negative):


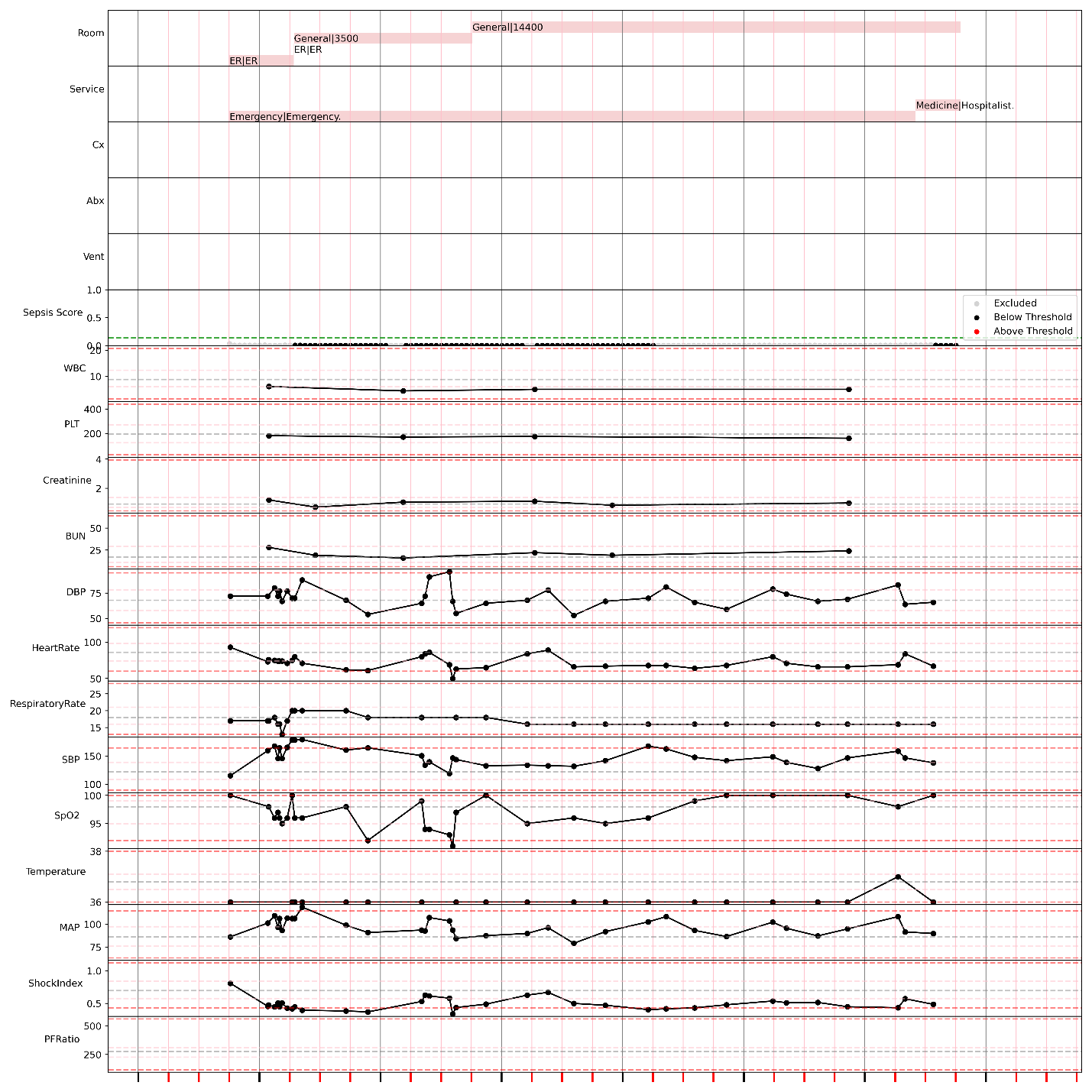


This patient was never septic, and the alert never crossed the threshold.

Example of alert failure (false positive):


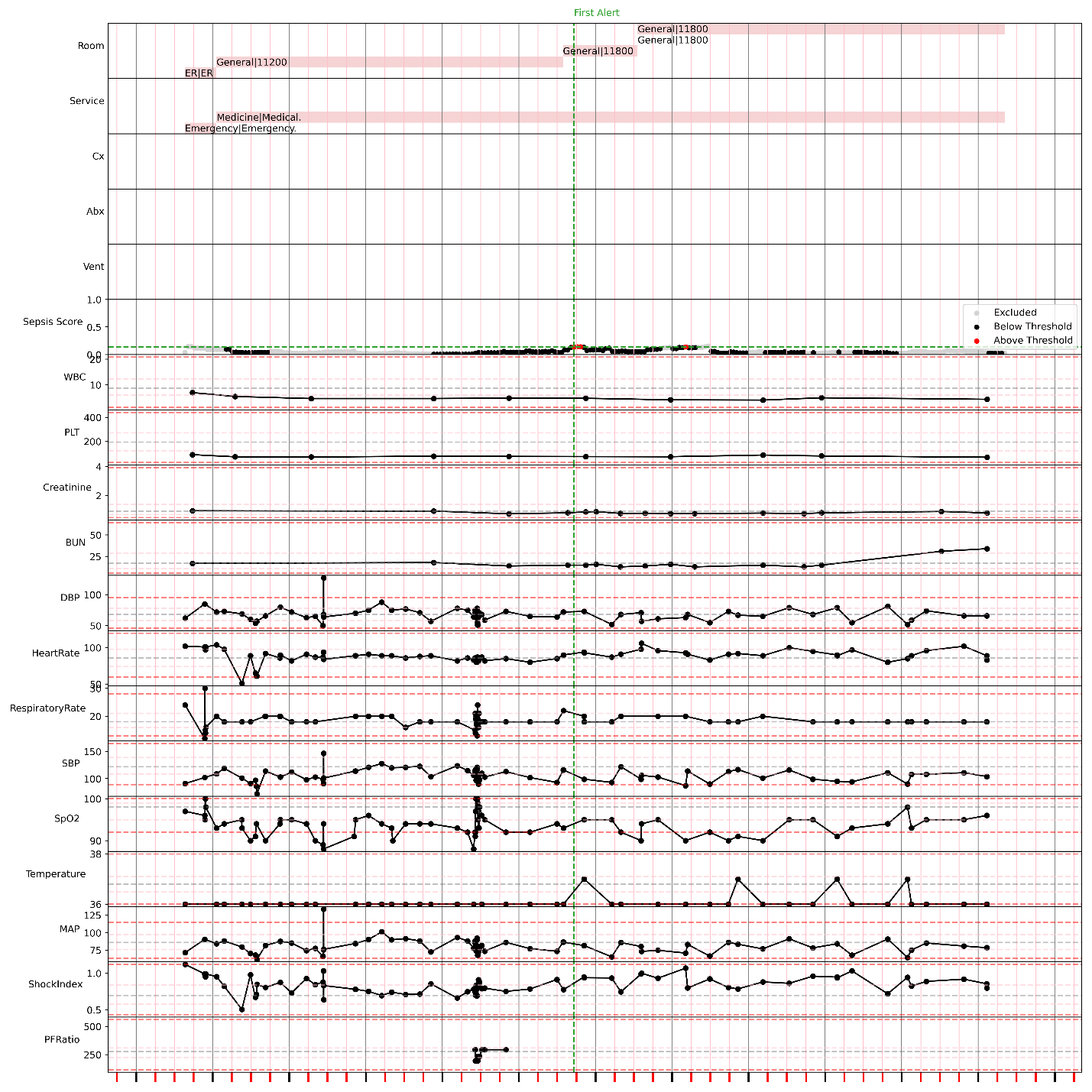


While the patient was never septic, there were short periods where the score just barely exceeded the threshold.

Example of model failure (false positive):
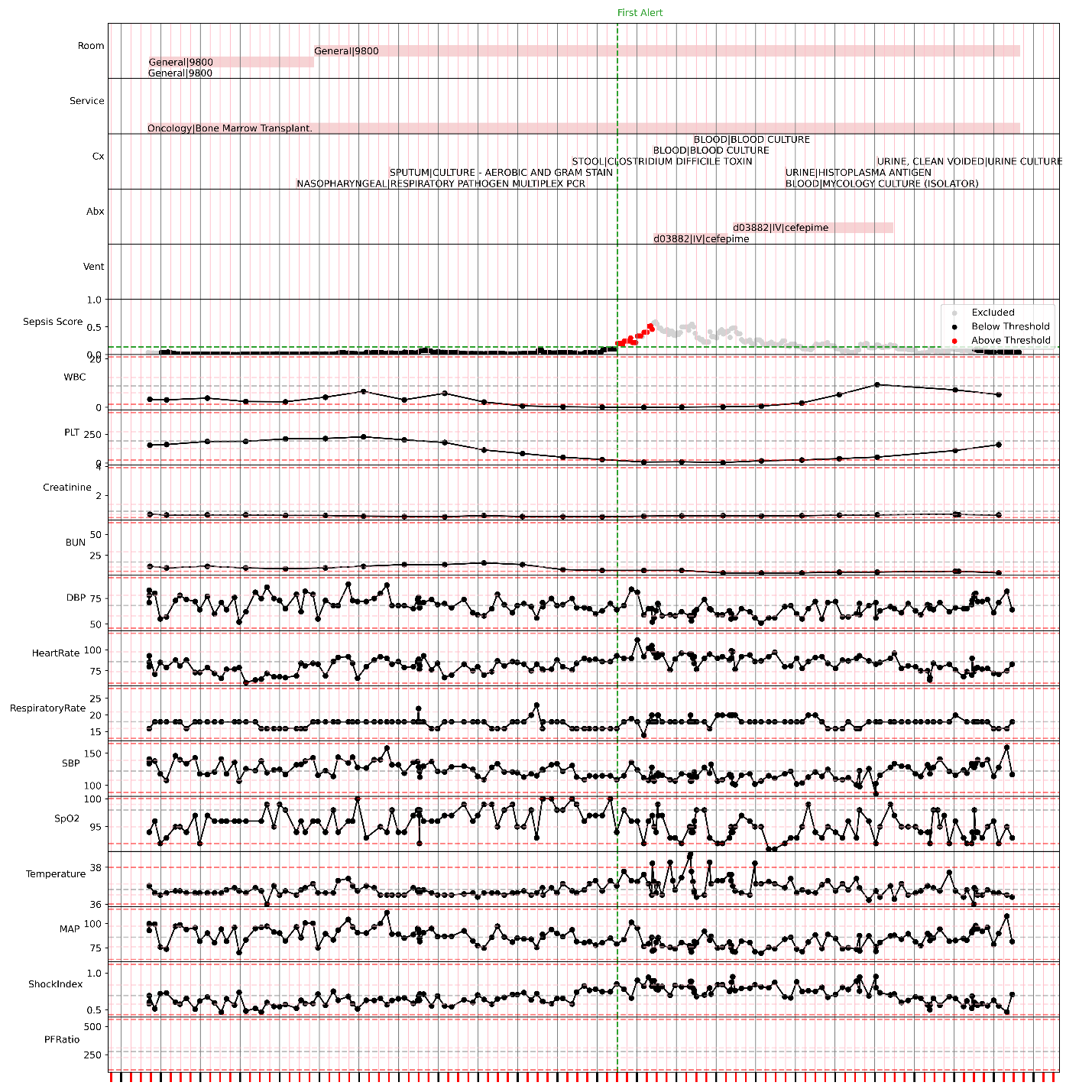


While this patient was never septic (due to not meeting qSOFA), they did receive sepsis-relevant anti-infectives and cultures.
